# Cross-linked Chitosan-Based Shell with Mirtazapine Lipid Polymer Hybrid Core as Integrated Spray-Dried Bionanocomposites for Boosted Brain-Directed Oral Delivery

**DOI:** 10.1007/s12035-025-05403-5

**Published:** 2025-12-08

**Authors:** Dalia M. Elbehairy, Enas Elmowafy, Rihab Osman, Omaima A. Sammour

**Affiliations:** Department of Pharmaceutics and Industrial Pharmacy, Faculty of Pharmacy, Ain Shams University, Monazzamet Elwehda Elafrikeya Street, P.O.B. 11566, Abbaseyya Cairo, Egypt

**Keywords:** Mirtazapine, Lipid polymer hybrid, Chitosan coating, Spray drying, Depression, Oral

## Abstract

Brain-directed delivery can elaborate locally to treat various brain ailments like the globally prevalent depression. Fulfilling such a goal via oral delivery can be conceivable owing to its expedience of self-medication and non-invasiveness. In this context, the oral behavioral trend was customized via tripolyphosphate cross-linked chitosan-based shell with lipid polymer hybrid core (TPP:CS-LPH) enclosing the antidepressant drug, mirtazapine (MIR). LPH nanoparticles were made of the PLGA core and the newly investigated lipid shell of lecithin and the pegylated lipid, Tefose 1500. Considering industrial scalability and applicability, spray drying was performed with thorough characterization. The impact of a cross-linked chitosan-based shell with LPH core on the in vivo pharmacokinetic behavior in brain and blood and pharmacodynamics in stressed mice (either daily regimen or every 3 days) was assessed and correlated to daily oral MIR suspension. The coated spray-dried nanocomposites were felicitously formed, exhibiting > 95% MIR association efficiency, controlled pH-dependent MIR release, and physicochemically, structurally, and morphologically verified cross-linked CS coating. For the selected formula (SD/TPP:CS-LPH_o_), the cross-linked chitosan-based shell yielded a 39-fold increase in brain *C*_max_ and extended *t*_1/2, brain_ equivalent to 4.7 times compared to oral MIR suspension. The superior antidepressant efficacy of SD/TPP:CS-LPH_o_, especially when administered every 3 days, was also accentuated in terms of considerable amelioration of behavioral response, decreased levels of brain biomarkers (brain-derived neurotrophic factor and serotonin), and histopathological findings. In conclusion, promising brain-directed oral delivery for MIR with improved bioavailability and therapeutic efficacy can be made possible by this tactic.

**Graphical Abstract:**

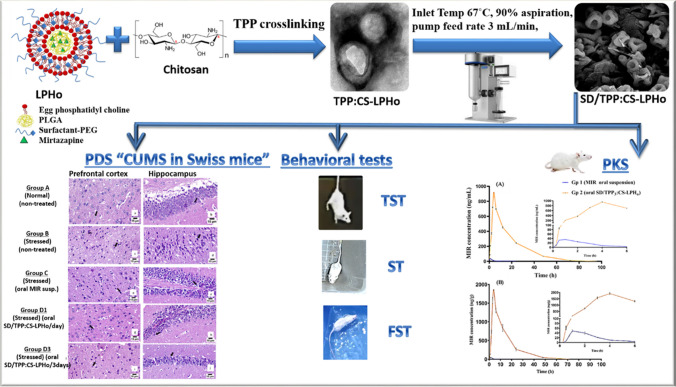

**Supplementary Information:**

The online version contains supplementary material available at 10.1007/s12035-025-05403-5.

## Introduction

From a mental and neurological public health perspective, the most critical burden facing the world’s population is chronic brain disorders and the related grim forecasts of an increase in the proportion of affected persons experiencing neurological ailments in the future [1]. Such issues arouse considerable solicitude for the headway of the intervention of functional neuropharmaceuticals [2]. However, the strict biological protective regulation that restricts drug access across the blood-brain barrier (BBB) presents great defiance. In such instances, moving to a “nanoscale delivery design methodology” seems attractive as the small-sized nanocarriers can act as facilitators of free entry to brain tissue.

Various non-invasive routes, outperforming the intravenous one, have been exploited for brain-directed delivery, for instance, intranasal as well as oral routes, considering the chronic nature of brain disorders and the necessity for long-term regularly administered treatments [3]. Of these, oral delivery is deemed the foremost alluring due to its compliance and expedited drug administration. An assortment of orally administered nanoplatforms enclosing neuropharmaceuticals including lipid-based nanocarriers, nanocrystals, and polymeric nanocarriers has been investigated, yielding maximized drug absorption, prolonged half-life, and stability in the gastrointestinal system [4–6].

Among the diversified nanoparticles (NPs), lipid polymer hybrid nanoparticles (LPHNPs) are exemplified as prospective nanoplatforms, consolidating traits of both polymeric and lipid NPs. They are considered core shell NPs with a polymeric core (e.g., PLGA and PLGA-PEG) encircled by a lipid monolayer (e.g., soybean lecithin and phosphoethanolamine-*N*-methoxy (polyethyleneglycol-2000)). Specifically, the beneficial leverage accomplished with interspersing polyethylene glycol (PEG) into LPHNPs can be ascribed to (i) the optimal extension of the nanocarriers circulation via its “dysopsonic” impact, averting the prompt reticuloendothelial system-mediated clearance; (ii) the increase of the particles’ water solubility, lessening the propensity for aggregation through steric stabilization; (iii) the inhibition of P-glycoprotein efflux (Pgp) which presents at the cerebrovascular endothelial cell membrane in brain tissue; and (iv) the assistance of rapid mucus penetration [7–9].

Accordingly, expedited orientation into the brain for the proposed pegylated LPHNPs can be contemplated. In this respect, herein, the increasingly utilized lipid, egg yolk lecithin (EPC), was combined with a pegylated lipid named Tefose 1500 (combined PEG-6 stearate and PEG-32 stearate) (TEF) as a newly investigated lipid mixture for the design of LPHNPs. With the aim of achieving customizable behavioral characteristics and mucoadhesive properties in gastrointestinal fluids, a polycationic carbohydrate biomacromolecule, chitosan (CS), was applied as a coating for the proposed LPHNPs. CS is a biopolymer that is non-immunogenic, non-carcinogenic, biocompatible, and biodegradable. CS coating was used, benefiting from its contribution towards enhanced cellular uptake and retention by opening tight junctions [10]. Considerably, to tackle the fast dissolution of the remaining unreacted CS and achieve tunable size and stability, a subsequent crosslinking with tripolyphosphate (TPP) has been attempted [11–14].

Building upon this tailored nanoplatform, an additional step of spray drying (SD) was performed aiming to form free-flowing dry powders with more enhanced industrial scalability [15]. Therefore, the target of this investigation is to combine LPHNPs design, CS coating, TPP crosslinking, and SD technique in an oral delivery system enclosing the antidepressant drug, mirtazapine (MIR), for the treatment of the globally prevalent brain disorder, depression. Such an approach can be auspicious for MIR delivery overcoming its poor water solubility (BCS class II drug), significant hepatic metabolism, 50% oral bioavailability, and 80% protein binding [16]. The physicochemical, morphological, thermal, and structural features of the proposed spray-dried nanosystem, as well as in vitro MIR release, were thoroughly assessed. Finally, in order to address their transit to the brain, the pharmacokinetics and pharmacodynamics behaviors of the proposed orally administered system were contrasted to the MIR suspension in male Swiss mice.

## Materials and Methods

### Materials

Acetonitrile (HPLC grade) and ammonium acetate are provided by Merck, Darmstadt, Germany. Chitosan (CS), viscosity 9 cp, and degree of deacetylation of 96% are kindly provided by Primex, Iceland. Diclofenac sodium (Internal Standard; IS) is provided by EPICO (El-Asher of Ramadan city, Egypt). Dimethyl sulfoxide (DMSO), sodium tripolyphosphate (TPP), sodium cholate (SC), and hematoxylin and eosin stain (H&E) are provided by Sigma-Aldrich, UK. Egg yolk lecithin (phosphatidyl choline) (EPC) is provided by Lipoid Company, Germany. Mirtazapine is provided by Megafine Pharma, India (kindly supplied by AUG Pharma, Cairo, Egypt). Poly(lactic-co-glycolic acid) (PLGA) copolymer, PURASOR PDLG (DL-lactide and glycolide), and 5002 A (50/50DL-lactide/glycolide copolymer acid terminated) are provided by Purac Biomaterials Gorinchem, The Netherlands. Tefose 1500 (TEF, mixture of PEG-6 stearate, and PEG-32 stearate) is provided by Gattefosse, France. Ultrapure water (obtained using MilliQ plus) is provided by Millipore Iberica, Spain. Glacial acetic acid, hydrochloric acid, mannitol, potassium dihydrogen phosphate, and disodium hydrogen phosphate are provided by El-Nasr Pharmaceutical Chemicals, Abo Zaabal, Cairo, Egypt.

### Preparation and Optimization of Mirtazapine-Loaded Lipid Polymer Hybrid Nanoparticles (MIR-LPHNPs)

MIR-LPHNPs were formulated employing the nanoprecipitation self-assembly technique. Briefly, accurately weighed amounts of PLGA (polymer) and MIR were dissolved in 1 mL acetone. Egg yolk lecithin/Tefose 1500 (EPC/TEF) in a 1:1 mass ratio and sodium cholate (SC) (0.07% w/v) were dissolved in water containing 4% v/v ethanol heated up to 60 °C to ensure complete lipid dissolution [17]. The organic phase was dripped on the stirred aqueous phase dispersion at a phase volume ratio of 1:2. Stirring the dispersion at 100 rpm and room temperature was continued for approximately 4 h until the evaporation of the organic solvent.

Optimization of MIR-LPHNPs was performed based on a *Box-Behnken design* (BBD) while applying the values obtained from a preliminary study. The selected critical material attributes (CMAs) were PLGA, lipids, and MIR concentrations and were assigned codes A, B, and C, respectively. Three levels were investigated for each variable, low (−1), medium (0), and high (1), as follows: PLGA concentration at 1, 2, and 3 (%w/v), lipid concentration (as a percent of PLGA used) at 6.7, 13.35, and 20 (%w/w), and MIR concentration (as a percent of PLGA used) at 5, 10, and 15 (%w/w).

Response surface methodology (RSM) helped to evaluate the effect and interactions of the chosen variables on *Y*_1_: particle size (PS), *Y*_2_: encapsulation efficiency (EE %), and *Y*_3_: zeta potential (*ζ*), which were the chosen responses (critical quality attributes CQAs) for the design. The desired quality target product profile (QTPP) was PS less than 100 nm and maximum EE%. A total of 17 practical runs were proposed by BBD, coded from F1 to F17. Five runs were used as checkpoints to validate the design-generated equations,

(Table 1), and the % bias was calculated according to Eq. (1). The optimized formula (LPH_o_) was recommended by the implemented design.

**Table 1:** Codes and composition of all experimental runs (formulae) for the preparation of MIR-loaded LPHNPs according to Box-Behnken response surface methodology

**Run**	**Code**	**A**	**B**	**C**	**Experimental PS** ** ± s.d. (nm)**	**Predicted PS** **(nm)**	**Experimental** **EE ± s.d. (%)**	**Predicted EE (%)**	**Experimental** **ζ ± s.d. (mV)**	**Predicted ζ**
1	F_1_	2.00	20.00	5.00	113.40 ± 4.45	108.70	86 ± 2.83	85.58	−19 ± 2.33	−18.33
2	F_2_	2.00	13.35	10.00	114.80 ± 4.45	113.42	57 ± 1.41	55.60	−11.9 ± 0.07	−11.50
3	F_3_	2.00	20.00	15.00	130.00 ± 0.42	140.34	46 ± 2.82	45.33	−14.7 ± 0.14	−15.24
4	F_4_	2.00	6.70	15.00	120.60 ± 0.63	125.30	54 ± 1.41	54.42	−10.9 ± 2.64	−11.57
5	F_5_	2.00	13.35	10.00	117.60 ± 3.45	113.42	56 ± 2.83	55.60	−10.9 ± 0.77	−11.50
6	F_6_	3.00	20.00	10.00	290.00 ± 3.61	297.15	56 ± 2.83	54.63	−3.97 ± 0.26	−4.11
7	F_7_	2.00	13.35	10.00	113.00 ± 4.45	113.42	54 ± 1.41	55.60	−12 ± 1.75	−11.50
8	F_8_	2.00	6.70	5.00	108.80 ± 0.14	98.46	60 ± 1.41	60.67	−17.1 ± 0.77	−16.56
9	F_9_	3.00	6.70	10.00	269.00 ± 1.84	281.79	50 ± 7.07	47.53	−1.68 ± 0.21	−1.69
10	F_10_	3.00	13.35	5.00	277.60 ± 1.34	275.15	54 ± 2.83	55.80	−2.9 ± 1.20	−4.13
11	F_11_	1.00	13.35	15.00	82.29 ± 0.93	84.74	47 ± 3.82	44.20	−13.1 ± 1.56	−10.97
12	F_12_	1.00	6.70	10.00	72.00 ± 1.57	64.85	57 ± 2.82	58.38	−12.4 ± 1.26	−12.26
13	F_13_	1.00	20.00	10.00	87.56 ± 9.08	74.77	65 ± 2.27	67.08	−15.3 ± 0.35	−15.29
14	F_14_	1.00	13.35	5.00	79.00 ± 0.76	96.50	87 ± 3.53	84.95	−17.1 ± 0.42	−17.78
15	F_15_	2.00	13.35	10.00	112.00 ± 4.45	113.42	55 ± 2.83	55.60	−10.8 ± 0.07	−11.50
16	F_16_	3.00	13.35	15.00	362.90 ± 11.95	345.41	48 ± 4.24	50.05	−3.56 ± 0.07	−2.88
17	F_17_	2.00	13.35	10.00	109.70 ± 2.45	113.42	56 ± 2.83	55.60	−10.8 ± 0.07	−11.50
Checkpoint 1	F_18_	1.00	8.00	7.00	72.50 ± 0.09	72.65	65.5 ± 2.54	67.72	−15.1 ± 0.56	−15.05
Checkpoint 2	F_19_	1.00	20.00	7.00	87.97 ± 0.02	83.86	85.4 ± 4.56	89.01	−16.5 ± 1.42	−17.86
Checkpoint 3	F_20_	1.50	10.50	5.00	88.07 ± 0.89	86.54	73.6 ± 3.34	69.71	−18.4 ± 1.06	−17.88
Checkpoint 4	F_21_	1.50	15.00	9.00	85.14 ± 0.54	90.63	59.3 ± 4.34	62.28	−14.8 ± 0.14	−14.08
Checkpoint 5	F_22_	2.80	15.00	10.00	222.0 ± 11.31	221.36	54.4 ± 4.53	52.95	−4.62 ± 0.36	−4.47
Optimized	LPH_o_	1.00	15.39	5.00	82.24 ± 0.19	79.97	87.31 ± 2.35	89.82	−17.90 ± 1.34	−18.21

A, PLGA concentration (%w/v), the coded levels (−1), (0), and (+1) denote 1%, 2%, and 3%, respectively; B, lipid concentration as a percent of the polymer (%w/w), the coded levels (−1), (0), and (+1) denote 6.7%, 13.35%, and 20%, respectively; C, MIR concentration as a percent of the polymer (%w/w), the coded levels (−1), (0), and (+1) denote 5%, 10%, and 15%, respectively

^*^Average of three determinations. s.d., standard deviation; PS, particle size; EE, encapsulation efficiency; ζ, zeta potential

1 $$\%Bias={\vert Predicted-Actual\vert}/{\vert actual\vert}\times100$$

### *Preparation and Optimization of TPP Cross-Linked CS-Coated LPH*_*o*_

CS-coated LPH_o_ (CS-LPH_o_) was obtained based on the deposition of the positively charged CS on the negatively charged surface of the prefabricated optimized MIR-LPHNPs. Briefly, CS-LPH_o_ was formed by adding the previously prepared LPH_o_ dropwise to a 0.2% w/v CS solution in 0.5% (v/v) acetic acid under continuous magnetic stirring at 100 rpm for 2 h at 25˚C [18]. An aqueous TPP solution, prepared at different concentrations namely 0.06%, 0.1%, 0.2%, and 0.33% w/v, was then added dropwise using a syringe in a volume ratio of 2.5:1 (CS-LPH_o_:TPP) [19] under continuous magnetic stirring at 100 rpm for 2 h at 25 °C. The prepared coated NPs were characterized as will be described later.

### *Spray Drying of TPP Cross-Linked CS-LPH*_*o*_

Mannitol (Man) was used as a drying protectant during spray drying to prepare powder for oral delivery [20]. Accordingly, Man was added to the fresh TPP:CS-LPH_o_ dispersion in a concentration of 25% w/w of the solid content, and the total solid final concentration was kept at 0.4% w/v in feed solutions. The nano-dispersions were spray dried using a Buchi mini spray dryer (Mini B-290: Buchi Laboratorium, AG, Switzerland) at an air flow rate of 400 L/h. The pump feed rate of 3 mL/min, the inlet temperature of 67 °C, and the aspiration rate of 90% were the utilized spray drying parameters. The temperature at the outlet, as a result, varied between 35 and 37 °C. The spray dried formulae were coded with a prefix SD.

### In Vitro* Characterization of MIR-LPHNPs and TPP:CS-LPH*_*o*_

#### Particle Size (PS), Polydispersity Index (PDI), and Zeta Potential (ζ)

PS, PDI, and ζ of MIR-LPHNPs and TPP:CS-LPH_o_ were determined utilizing a PS analyzer relying on dynamic light scattering (DLS), Zetasizer® Nano-ZS (Malvern instruments, Malvern, UK). The analysis was carried out as previously reported in the literature [17].

#### Determination of MIR Encapsulation Efficiency (EE%)

By quantifying the amount of MIR entrapped inside the formed LPHNPs, the drug EE% was estimated directly. MIR-LPHNPs dispersion was centrifuged at 30,000 rpm for 1 h at 4 °C utilizing a cooling ultracentrifuge (Hermle Z216, Germany). The washed pellets were then resuspended in water, frozen, and directly freeze-dried. A precisely weighed freeze-dried powder (5 mg) was dissolved in 1 mL DMSO, and MIR concentration was determined at *λ*_max_ (295 nm) using UV spectrophotometry double beam, model UV-1601PC (Shimadzu, Kyoto, Japan). The encapsulation efficiency (EE%) was determined using Eq. (2) [17].

2 $$EE\left(\%\right)=\frac{\text{Actual drug in NPs}}{\text{Initial MIR added}}\times100$$

#### Particle Examination Using High-Resolution Transmission Electron Microscope (HR-TEM)

Freshly prepared dispersions of the optimized MIR-LPH (LPH_o_) and selected TPP:CS-LPH_o_ were stained (1% phosphotungstic acid) and examined using TEM at 200 kV (JEM-1010) (JEOL Ltd, Tokyo, Japan) [17].

### Characterization of Spray-Dried Powder (SDP)

#### Powder Yield

Gravimetric method was used to determine the yield of the spray drying process as described earlier [21].

#### PS Determination

The volume PS distribution was determined utilizing a Malvern Mastersizer 2000® laser diffractometer (Malvern instruments, Malvern, UK) with the Aero S dry powder dispersion system using a dispersive air pressure of 4 bar [22]. The observed average PS was expressed as volume mean diameter (VMD) and the particles’ dispersity and uniformity were indicated as span. The diameters at 10, 50, and 90 and the volume mean diameter in µm were denoted by D[v,10], D[v,50], D[v,90], and D[4,3], respectively.

#### Determination of MIR Content

SDP drug content and drug association efficiency percentage (AE%) were determined as follows: an accurately weighed amount (10 mg) of SDP was added to 2 mL (75% v/v DMSO and 25% v/v acetic acid) and bath sonicated for 10 min at room temperature [23]. Utilizing UV spectroscopy, the drug content was estimated at the predetermined *λ*_max_. MIR association efficiency and theoretical and actual loading capacities were determined utilizing Eqs. (3), (4), and (5), respectively:

3 $$MIR\;association\;efficiency\;(AE)\;=\frac{\text{Actual MIR amount}}{\text{Theoretical MIR amount}}\times100$$

4 $$Theoretical\;MIR\;loading\;capacity\;=\frac{\text{Theoretical MIR amount}}{\text{Powder weight}}\times100$$

5 $$Actual\;MIR\;loading\;capacity\;=\frac{\text{Actual MIR amount}}{\text{Powder weight}}\times100$$

#### Recovery of Nano-dispersion from SDP

Nano-dispersion recovery from SDP was performed by suspending 10 mg of each spray dried formula in 1 mL of 0.1N HCl (pH 1.2) for 2 h, followed by raising pH to 7.4 by addition of phosphate buffer solution (1 M, pH 7.4) under mild stirring at 25 °C to a final volume of 10 mL. The PS was followed at different time intervals in both media. Recovery index (*R*_index_) was calculated as follows:

6 $$R_{index}=\frac{S_F}{S_I}$$

where *S*_F_ is the final PS recovered after SD and *S*_I_ is the initial PS before SD [24]. The recovered SD/TPP:CS-LPH_o_ after 10 min in 0.1N HCl was visualized using TEM.

#### In Vitro MIR Release Study

Using the previously mentioned dialysis membrane diffusion method, the in vitro drug release study was carried out, with minor modification [25]. The test was performed on SD/CS-LPH_o_ (without TPP crosslinking), SD/TPP_1_:CS-LPH_o_, SD/TPP_2_:CS-LPH_o_, and SD/TPP_3_:CS-LPH_o_. A precise amount of SDP (equivalent to 2.5 mg of MIR) was suspended in 1 mL of deionized water inside a dialysis membrane of MwCO: 12,000–14,000 Da (Rancho Dominguez, CA, USA). The membrane was then sealed at both ends and immersed in a capped vial containing 50 mL of 0.1N HCl. Samples were incubated in a shaking water bath (Kotterman, Hanigsen, Germany) at 37 ± 0.5 °C and 50 strokes/min.

At specified time intervals (0.5, 1, and 2 h), 1 mL samples were taken and replenished by an equivalent volume of 0.1N HCl. After 2 h, each dialysis bag was removed from 0.1N HCl and placed for 4 h in 50 mL of 0.1 M PBS (pH 6.8). Samples were taken at 3, 4, and 6 h and replenished with fresh buffer solution. After 6 h, each dialysis bag was removed and placed in 50 mL of 0.1 M PBS (pH 7.4). Samples were removed at 8, 24, 48, 72, 96, 120, 144, and 168 h and substituted with newly prepared buffer to keep sink conditions. The in vitro release of MIR powder treated as the SDP was also performed for comparison. The amount of MIR released was quantified spectrophotometrically at the respective *λ*_max_ values of 315 and 290 nm for HCl and PBS.

#### Moisture Content

Water content of selected SDP was examined by thermogravimetric analysis (TGA) (PerkinElmer, Pyris I-Wellesley, USA) as previously described [26].

#### Scanning Electron Microscope (SEM)

The shape and surface features of selected SDP were examined at 20 kV acceleration voltages using SEM (Quanta FEG 250 scanning electron microscope, Cambridge instruments, Cambridge, UK) [22].

#### Fourier Transform-Infrared (FT-IR) Spectroscopy

FT-IR spectra of MIR, PLGA, TEF, EPC, CS, and TPP, the selected SDP plain and medicated were detected with an FT-IR spectrometer (Shimadzu, Japan) utilizing the KBr disc method, and the spectrum was obtained from 4000 to 400 cm^−1^ at a resolution of 4 cm^−1^ [17].

#### Differential Scanning Calorimetry (DSC)

The thermal attributes of all individual components namely MIR, PLGA, TEF, EPC, CS, and TPP; the physical mixture of 1:1:1:1:1 of MIR:PLGA:EPC:TEF:TPP:CS; and the selected SDP plain and medicated were investigated using DSC TA-60 WS thermal analyzer (Shimadzu, Tokyo, Japan). Samples were heated to 250 °C at a heating rate of 10 °C per minute under nitrogen [26].

#### X-ray Powder Diffraction (XRPD)

The existence of crystalline and amorphous content of pure materials (MIR, PLGA, TEF, EPC, CS, Man, and TPP) and the selected SDP were determined using XRPD [26]. The samples were analyzed using a diffractometer (X-pert; Philips, Guildford, UK) operating at 30 mA, 45 kV, and scanning angles of 5–45°.

#### Stability Study

The effect of storage on the selected SD TPP cross-linked CS-coated LPH formula was evaluated by determination of VMD D[4,3] and AE % of dry powder after storing the formula for 1, 3, and 6 months in a silica desiccator at ambient conditions [27].

#### In Vivo Biological Evaluation

Male Swiss mice, weighing 20–25 g, were selected for in vivo studies. The Research Ethics Committee of the Faculty of Pharmacy at Ain Shams University in Cairo, Egypt, reviewed and approved the experimental procedures for all animal studies. The committee granted REC number 165. Prior to the experiment, the animals were placed in quarantine for a week in order to give them time to adequately adapt to the existing experimental surrounding.

#### Animal Housing

Male Swiss mice weighing 20–25 g were obtained from Abo Rawash, Giza, Egypt. Mice were kept in plastic cages with a 12-h light/dark cycle at room temperature (25 °C). They were fed on normal rodent chow and water ad libitum supplied by El-Nasr Pharmaceuticals Co., Egypt. The animals were quarantined under these conditions for 1 week before starting the experiment to allow for adaptation to the experimental environment.

#### Pharmacokinetic Study

Mice were divided randomly into 2 groups (*n* = 4 each time interval): Group 1 (Gp 1) received a volume of 0.5 mL oral free MIR suspension in distilled water in a dose equivalent to 10 mg/kg by oral gavage [28]. Gp 2 received oral SD/TPP_3_:CS-LPH_o_ in a dose equivalent to 5 mg/kg by oral gavage (0.5 mL).

Using heparinized Helmington microcapillary tubes (25 µL of 20 mM sodium heparin), blood samples withdrawn from the retro-orbital vein were centrifuged for 15 min at 9000 rpm (cooling centrifuge, Hermle Labortechnik GmbH, Z216MK, Germany) [17]. Sampling continued for 24 and 96 h post-administration of oral free MIR and oral SDP, respectively, based on the data obtained from a pilot study.

At each time interval, four mice were sacrificed, and the animals’ brains were dissected, then homogenized at 24,000 rpm for 2 min following twofold dilution with PBS (pH 7.4). Each brain homogenate (BH) and plasma sample was combined with an equal volume of acetonitrile, vortexed, and centrifuged for 10 min at 5000 rpm. Diclofenac sodium 100 ng/mL was used as an internal standard (IS). The supernatant organic layer was evaporated to dryness at 50 °C under nitrogen followed by reconstitution in the mobile phase (a mixture of acetonitrile and ammonium acetate, 0.01 M, 32:68 v/v; pH 4) and subsequent injection into a high-performance liquid chromatography (HPLC) apparatus (PerkinElmer, California, USA). MIR content was quantified at a wavelength of 295 nm, employing a validated HPLC method [29].

Mean MIR concentrations in BH and plasma samples were plotted versus time. From the attained plots, the following pharmacokinetic parameters were computed: (i) peak concentrations (*C*_max plasma_ and *C*_max brain_), (ii) the time required to reach *C*_max_ (*t*_max plasma_ and *t*_max brain_), (iii) the area under the MIR concentration-time curve (AUC_plasma_ and AUC_brain_), (iv) the mean residence time (MRT_plasma_ and MRT_brain_), and (v) the time to reach half plasma and homogenate concentration (*t*_1/2plasma_ and *t*_1/2brain_).

### Pharmacodynamic Study

#### Induction of Depression in Mice by Adopting Chronic Unpredictable Mild Stress (CUMS) Protocol

The 14-day CUMS experimental protocol was employed by applying a range of stressors at random times daily to reduce the probability of stress prognosis [30]. The animals were split into two groups: non-stressed (negative control, *n* = 5) and stressed (CUMS, *n* = 20) mice. Because of the stress odor, groups of stressed animals and the negative control were separated in different rooms.

The animal was subjected to stress by swimming in an open, cylindrical container (height of 25 cm and diameter of 10 cm). The water within the container was kept at either 25 ± 1 °C or 12 ± 1 °C for the “cold swim.” By turning on the lights for 12 h, the light/dark cycle was reversed. After then, the lights were off for another 12 h. With the cage angled at 45°, wood shavings were wetted using water (200 mL/100 g wood shaving bedding). As reported previously, a clothespin positioned one centimeter from the tail’s base was used to accomplish the tail squeeze [30].

#### MIR Administration

Mice were randomly divided into five groups (*n* = 5) as follows: Gp A: normal mice, normal non-stressed group did not receive any treatment. Gp B: stressed group and received no treatment, (stressed, non-treated). Gp C: stressed group, received oral MIR suspension by oral gavage in distilled water, at a drug dose of 5 mg/kg. Gp D_1_: stressed group, received SD/TPP_3_:CS-LPH_o_ via oral gavage, reconstituted in distilled water, at a dose of 5 mg/kg daily. Gp D_3_: stressed group, received SD/TPP_3_:CS-LPH_o_ via oral gavage, reconstituted in distilled water, at a dose of 5 mg/kg every 3 days. Gps C and D_1_ were administered daily, from day 8 to 14 while Gp D_3_ was administered on days 7, 10, and 13 (every 3 days) [31].

On day 15, 24 h after the administration of the last dose, behavioral tests were performed for all tested groups. After that, decapitation was carried out, and their hippocampal and prefrontal cortex of the brain was harvested for biochemical analysis as previously stated [30]. For histological examination, brains were removed and dissected on ice for PFC isolation [32].

### Behavioral Tests

#### Forced Swimming Test (FST)

Each mouse was made to swim up to a height of 15 cm in a 30 × 15 × 30 cm^3^ (L × B × H) water-filled vessel at 23 ± 2 °C. Mice would enthusiastically swim in the water after they were submerged because they were afraid of drowning. Despite their best efforts, mice were unable to break free from the cylinder, which caused them to become hopeless and appear as if they were floating on the water’s surface. Following the first 2 to 3 min of intense activity, they displayed a period of immobility by moving very little. When an animal is passively floating in the water with its face above the surface and its body slightly bent but erect, it is said to be immobile. On day 15, the time of immobility was recorded for a duration of 6 min [33].

#### Tail Suspension Test (TST)

As previously depicted, the total amount of time that tail suspension caused immobility was determined [34]. Using adhesive tape positioned around 1 cm from the tail tip, mice that were acoustically separated were suspended 50 cm above the ground. Mice are regarded as immobile when they are hanging motionless and inert. Likewise, in the FST, during a 6-min period, the observed immobility time was estimated.

#### Splash Test (ST)

This investigation was carried out exactly as previously stated [33]. The procedure involves misting a mouse’s dorsal coat with a 10% sucrose solution while it is placed individually in a transparent box of dimensions 9 × 7 × 11 cm^3^. The viscosity of the sucrose solution causes the mouse fur to become dirty, prompting the animals to begin grooming. After that, within a 5-min period, grooming time was recorded as a measure of motivational behavior and self-care, being comparable to the apathetic symptoms of depression.

#### Biochemical Marker Analysis

The dissected hippocampi and prefrontal cortices (PFCs), after mice decapitation, were homogenized in 0.1 M phosphate buffer (pH 7.4) forming a 10% (w/v) homogenate. After centrifuging the homogenates for 15 min at 4 °C and 4000 rpm, aliquots of the supernatants were separated and used for neurochemical analysis [30]. The brain-derived neurotrophic factor (BDNF) and serotonin (5-HT) were determined using ELISA techniques described elsewhere [35]. ELISA kits for BDNF (Sigma, MBS824814) were of sensitivity 0.5 pg/mL and standard curve range, 31.2–2000 pg/mL. Moreover, ELISA kits (Sigma, MBS2611553) used for 5-HT determination were of sensitivity 0.5 ng/mL and standard curve range, 1.56–100 ng/mL. It is to be noted that the data were expressed as pg/g in brain tissues for BDNF and as ng/g in brain tissues for 5-HT. The effect of depression induced in the CUMS protocol was evaluated in terms of measurement of both BDNF and 5-HT levels in PFC and hippocampus. The % decrement in each marker level was calculated for each gp as the difference between biomarker level if stressed, non-treated, and that of each gp.

#### Brain Histopathological Examination

The autopsied brain samples were preserved in 10% formol saline for 24 h, treated as previously described and examined using light microscopy [32].

### Statistical Analysis

For the in vitro results, the mean of three determinations ± standard deviation (sd) was used to express data. Using the GraphPad Instat software, the mean values were compared using the Student *t* test or an ANOVA. At *p* ≤ 0.05, differences were deemed statistically significant. ANOVA was utilized to evaluate the significance of the model after each response (PS, EE%, or *ζ*) from the BBD design recommended by *design expert 7.0 aprobado* after being fitted to a complete quadratic equation. Statistical analysis was carried out using ANOVA followed by Tukey’s multiple comparison test for the mean values comparison. Pharmacokinetic analysis data was expressed as mean ± standard error (SE). Non-compartmental analysis using PKSolver Excel Add-in was used for pharmacokinetic analysis.

## Results and Discussion

### Characterization of MIR-LPHNPs

#### Statistical Analysis and Modeling

MIR-LPHNPs were prepared using 4% v/v ethanol in the aqueous phase, a 1:1 lipid ratio of EPC:TEF, and SC concentration of 0.07% w/v based on a preliminary study (*data not shown*). Addition of SC was a prerequisite in the formulation to ensure MIR encapsulation. A 3^3^ design was then built up for MIR-LPH optimization relying on varying the concentrations of PLGA, lipid, and MIR. The limits fed for each CMA were used to construct the design in space and build the three-dimensional cube vertices.

The “Box-Cox” transformation recommended inverse square root transformation for PS and EE% responses (*λ* = −0.5) as the optimal lambda value for both responses was not within the 95% confidence interval (CI) range of the initial lambda (*λ* = 1) (Fig. S1: A & B). As for *ζ* response, no transformation was recommended as the original lambda (*λ* = 1) lay within the (0.39–1.28) range of the best lambda value (Fig. S1: C).

The regression analysis developed statistical models using the experimental data for MIR-PS, EE%, and *ζ* values (Table 1). For each response, the coefficient of determination (*R*^2^) was determined to be > 0.9 (Table S2), demonstrating both the high predictability and the satisfactory fit of the proposed models to the experimental data.

Comparing the experimental values of the response with the predicted values in the “Predicted versus Actual” plot, a strong correlation was delineated in the case of PS, EE%, and *ζ* (Fig. S1: D, E, & F, Table S2) [36].

“Externally Studentized residuals” revealed no outliers between all of the fed data with normally distributed values (Fig. S1: G, H, and I) [37].

After fitting the PS, EE%, and *ζ* to a quadratic model with *p* < 0.0001, the resulting Eqs. (7), (8), and (9) are displayed in Table S2. It is evident from Eq. (7) (Table S2) that the PS was significantly heightened by increasing PLGA concentration (Fig. S2-A). This can be exemplified by the different coefficients of *A* and *A*^2^, with *A* possessing a modest positive coefficient and *A*^2^ exhibiting a substantial negative coefficient, signifying a non-linear relationship. It is to be noted that PLGA concentration was the most influential factor. The increased viscosity of the resulting dispersion may be the cause of the notable increase in PS at higher PLGA concentrations [38]. This increased viscosity could either slow down the rate at which the organic solvent evaporates, producing larger PS, or resist the dissociation of the droplets into smaller particles, and hence reduce the impact of the shear force created by stirring [39].

In case of MIR, due to the high positive and the small negative coefficients of *C* and *C*^2^, respectively, increasing MIR concentration was associated with an initial decrease in PS followed by a sharp increase at high MIR levels. Incorporating higher MIR concentrations resulted in larger PS values, which may be due to either drug encapsulation within the NPs or drug interaction with the lipid layer surrounding the NPs [40]. Similar to how drug and polymer affected the PS, higher lipid concentrations significantly increased the PS. Earlier works reported the deposition of a multilayer of lipids on NPs upon using high lipid concentration or working with zwitterionic lipids only, impacting their size enlargement [41].

**Fig. 1:**
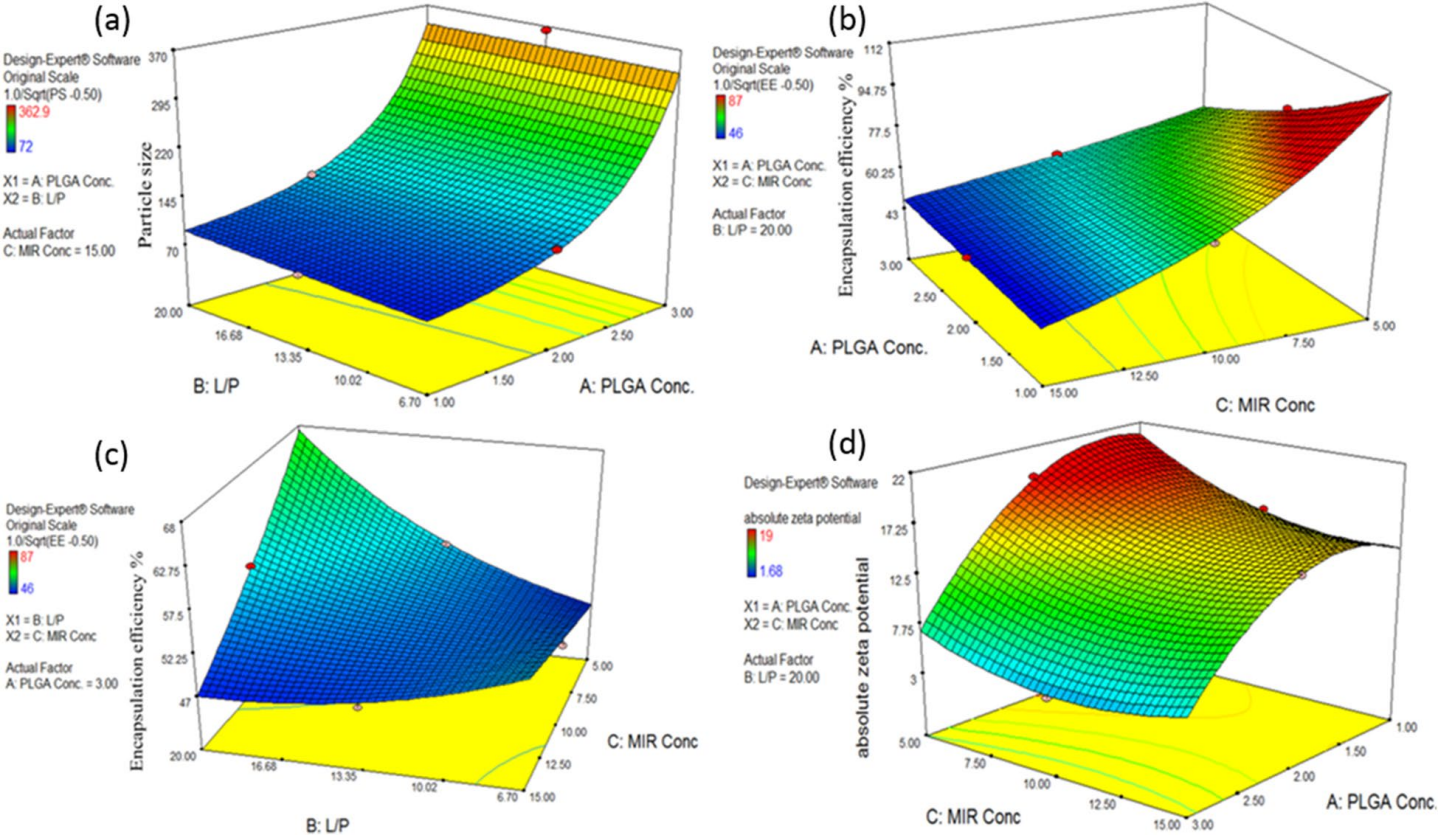
3D surface plots of** a** AB interaction between PLGA and L on the PS at 15%w/w MIR concentration, **b** AC interaction between PLGA and MIR on the EE% at 20%w/w L concentration. **c** BC interaction between L and MIR on the EE% at 3%w/v PLGA concentration and **d** AC interaction between PLGA and MIR on absolute zeta potential at 20%w/w L concentration

The interaction between PLGA and lipid concentrations (AB) on PS is illustrated in Fig. 1a. The increased lipid concentration decreased the effect of PLGA on increasing the PS, resulting eventually in PS values lower than expected as manifested by the negative AB coefficient in the BBD generated equation. At optimum PLGA and EPC concentrations, more compact small-sized LPHNPs are formed.

From Eq. (8), we deduced that PLGA concentration effect on EE% was the most prominent due to its highest coefficient. Increasing PLGA concentration was associated with a decrease in EE% most probably due to an increase in the concentration of polymer bounded by hydrophobic bond to the lipid in the particles’ core, leaving less free space for drug encapsulation; similar results were reported in previous studies [42].

Factor C (MIR concentration) exhibited a negative effect on EE%. As mentioned above, it is difficult to encapsulate cationic drugs into LPHNPs, and sometimes, their presence causes the failure of the formation of NPs. Poor encapsulation was probably due to a sort of formation of an ion-pair complex between the anionic PC of EPC and the cationic drug (MIR) opposing the availability of free drug for encapsulation during the self-assembly of LPHNPs. This ion-pair complex is most probably the reason for the increased PS observed with increasing MIR concentration. Some researchers overcame this problem by replacing some of the PC with cationic repelling lipids, thus breaking the ion-pair complex between MIR and EPC [9]. However, in our work, the inclusion of SC in a specified amount (being smaller in size than PC molecules) led to the formation of a lipophilic ion-pair complex, less soluble in the aqueous media, resulting in an improvement in EE% (Omar et al., 2020).

Investigating the significant interactions between the tested factors revealed that the AC interaction (PLGA-MIR) possessed a positive effect on EE% (Fig. 1b). At high PLGA concentration, more matrices might have been created to entrap the drug, possibly opposing the negative impact of MIR on EE%. The reverse was observed with the BC (lipid-MIR) interaction (Fig. 1c). The negative effect of MIR on EE% was intensified by high lipid concentration where the lipid had saturated the core by interacting with PLGA expelling MIR outside the particles, leading to the observed sharp decrease in EE%. Therefore, we can say that in order to formulate LPHNPs that can encapsulate appreciable drug amounts, an adequate lipid and PLGA concentration is required [9].

Equation (9) shows the highest coefficients for *A* and *A*^2^, indicating the commanding influence of this factor on *ζ*. From the main effect plot (Fig. S2: C, F and I), as PLGA concentration increases, *ζ* of LPHNPs was found to increase initially followed by a decrease at higher PLGA concentrations as manifested by the negative *A*^2^ coefficient. Increasing PLGA concentrations are associated with the orientation of lecithin (EPC) to hydrophobically interact with PLGA, leaving less free negative surfaces. Their hydrophilic head would extend to the external aqueous surrounding, forming a homogenous lipid-PEG (TEF) stabilized LPHNPs, while their hydrophobic tail would interact with the produced polymeric core to cause the lipids to self-assemble around it [42, 43].

In contrast, the lipid concentration increases (factor B), i.e., more total lipids relative to the polymer, resulting in an increase in *ζ* (Fig. S2-F) as noticed from the negative *B* and positive *B*^2^ coefficients. Indeed, the negative value of the surface charge was due to the presence of the negative phosphate group in the phospholipid (EPC) [9]. An additional parameter that significantly influenced the modulation of colloidal stability was the lipid-PEG density. Lipid-PEG chains may provide steric stabilization across the nanoparticle interface, preventing aggregation and enhancing the stability of the formulations of nanoparticles [9].

Concerning the MIR effect, it mainly decreased *ζ*, as manifested by its negative coefficient in Eq. (9). This effect is mainly due to the drug’s positive charges offered by its quaternary amino groups. Similar results were elucidated when incorporating fluoxetine (of cationic nature) inside LPHNPs. It was reported that the enclosed cationic drug caused a significant decrease in *ζ* of LPHNPs, which is made up of PLGA and soybean lecithin [44]. At high MIR concentrations, and after saturating the PLGA core, the drug’s effect was mainly located at the surface shielding the groups that imparted the negative *ζ* and eventually decreasing the *ζ* value.

Figure 1d delineates that the significant AC interaction (PLGA-MIR concentrations) caused a decrease in the effect of PLGA on lowering the *ζ*, resulting eventually in *ζ* values higher than expected as manifested by the positive AC coefficient in the BBD-generated equation.

#### Check Point Analysis

Five MIR-LPH dispersions at random were created, and their compositions are listed in Table 1. Equations (7–9) were utilized to compute the experimentally determined PS, EE%, and *ζ*. Their respective % bias, calculated using Eq. (1), ranged among 0.21 ± 0.12 to 6.44 ± 0.68% for PS, 2.66 ± 0.65 to 5.28 ± 0.915% for EE%, and 0.26 ± 3.73 to 7.92 ± 1.38% for *ζ*, revealing that all the bias % values were below 10% which confirmed the validation of the constructed model.

#### Selection of the Optimized Formula

By adjusting PS response to be in the range of 72–100 nm while EE% and ζ responses to maximum, the obtained desirability was 1. The suggested formula with the following composition, PLGA concentration of 1%, lipid concentration of 15.39% relative to the polymer amount, and MIR concentration of 5%, was prepared and coded LPH_o_.

#### ***TPP Cross-linked CS-Coated LPH***_***o***_*** (TPP:CS-LPH***_***o***_***)***

Upon adding TPP at 0.06% w/v, a significant increase in PS from 282.0 ± 5.02 to 332.4 ± 2.89 nm (*p* < 0.0001) was observed (Table 2). This was accompanied by a significant decrease in PDI and *ζ* from 0.333 ± 0.03, 47.5 ± 0.84 mV to 0.282 ± 0.011, and 37.4 ± 0.42 mV, respectively (*p* < 0.0001). The significant decrease in *ζ* delineated the decline in the number of available protonated CS amino groups due to its cross-linking with TPP [19].

**Table 2:** Effect of TPP concentration on the characteristics of TPP cross-linked CS coated LPH_o_

F-code	TPP conc. (% w/v)	PS^a^±sd (nm)	PDI^b^ ±sd	ζ^c^ ±sd (mV)
CS-LPH_o_	0	282.0±5.02	0.333±0.03	47.5±0.84
TPP_1_:CS-LPH_o_	0.06	332.4±2.89***	0.282±0.011***	37.4±0.42***
TPP_2_:CS-LPH_o_	0.1	304.3±2.05 ***	0.271±0.07***	29.0±0.42***
TPP_3_:CS-LPH_o_	0.2	263.5±7.14 ***	0.435±0.014***	16.1±0.212***
TPP_4_:CS-LPH_o_	0.33	ND		

All results are expressed as means of 3 determinations ± s.d. ^a^particle size, ^b^polydispersity index and ^c^zeta potential. Composition of CS coated optimized formula: 1%w/v PLGA concentration, 15.39%w/w lipid concentration, 5%w/w MIR concentration and 0.2%w/v CS. All formulae were obtained at volume ratio CS-LPH_0_: TPP 2.5:1. Statistical analysis was carried out using ANOVA followed by Tukey^’^s multiple comparison test to compare the data of different TPP conc. to 0% TPP. ^***^: *p*< 0.001. ND: not determined due to aggregates formation.

Upon increasing TPP concentration to 0.2% w/v, a significant decrease in PS and *ζ* from 332.4 ± 2.89 nm, 37.4 ± 0.42 mV to 263.5 ± 7.14 nm, and 16.1 ± 0.212 mV, respectively, (*p* < 0.0001) was noticed (Table 2). TPP cross-linking led to a PS reduction and a modification in the amount of positive charge on the surface, which may have decreased *ζ* [45].

All cross-linked nano-dispersions were positively charged, ensuring the presence of CS at the surface of all NPs formed, regardless of the extent of the cross-linking [19]. Noticeably, at 0.06 and 0.1%w/v TPP, there was a significant decrease in PDI (0.282 ± 0.011 and 0.271 ± 0.07, respectively) compared to 0% w/v TPP (0.333 ± 0.03), *p* < 0.0001. Further increase in TPP conc. to 0.2% w/v resulted in a significant increase in PDI (0.435 ± 0.014), (*p* < 0.001). At a TPP conc. of 0.33%, aggregates were observed.

When adding TPP to preformed CS NPs, the observed phenomenon of an initial increase in particle size followed by a decrease and eventual aggregation can be explained by the ionic interactions between TPP and CS. An initial increase in particle size could be due to ionotropic gelation [46]. Since, TPP is a polyanion that can use electrostatic forces to bind with cationic chitosan molecules. TPP can bind to the chitosan molecules on the surface of preformed CS nanoparticles when it is added, which causes a more extensive polymer network to develop, resulting in an initial increase in the size of the particles. Moreover, this initial increase could be due to the ability of TPP to cross-link chitosan molecules and increase particle size by acting as a cross-linking agent.

The significant decrease in particle size upon increasing the amount of TPP could be due to compaction that may result from the increased cross-linking effect of TPP leading to the formation of a more compact structure with reduced particle size. As the particles become closer together, their total size decreases. The positive charges on the chitosan molecules can be neutralized by excess TPP, which decreases the electrostatic repulsion between particles and permits them to pack together [46].

Aggregation that occurred upon further increase in TPP could be due to excessive cross-linking that occurs when particles are joined by TPP bridges. Moreover, charge neutralization and decreased stability due to excess TPP can neutralize the positive charges on the chitosan molecules, which can cause the particles to become less stable and agglomerate because of a decrease in electrostatic repulsion [47].

Optimal TPP concentration is required since the degree of cross-linking and particle stability are likely to be balanced by an ideal TPP concentration, producing nanoparticles with the required characteristics [48].

#### Particle Morphology Using High-Resolution Transmission Electron Microscope (HR-TEM)

**Fig. 2:**
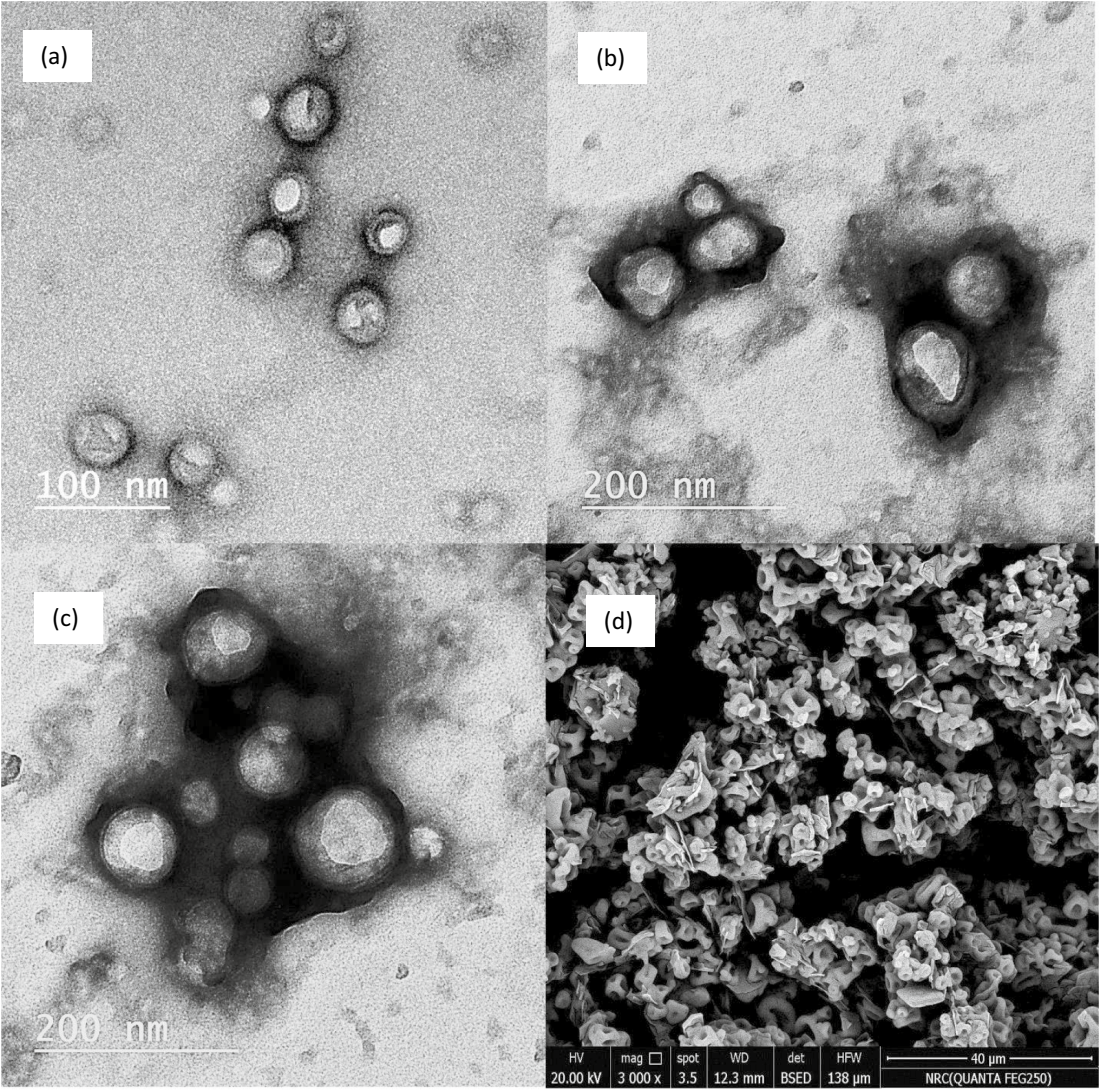
TEM images of **a** freshly prepared LPH_o_, **b** freshly prepared TPP_3_:CS-LPH_o_, **c** after recovery of SD/TPP_3_:CS-LPH_o_, 10 min following dispersion in 0.1N HCl, and **d** SEM image of SD/TPP_3_:CS-LPH_o_

TEM revealed that the freshly prepared hybrid particles (LPH_o_) possessed a spherical shape and an almost uniform size distribution (Fig. 2a) similar to what was observed previously for LPH [17]. TEM images of the freshly prepared TPP_3_:CS- LPH_o_ clarified the occurrence of a coating layer surrounding the particles that was less contrasted [49]. It is worth noting that CS coating and subsequent TPP cross-linking did not alter the spherical shape of LPH (Fig. 2b) [50].

#### Characterization of SDP

Exploring spray drying efficiency seems very crucial to guarantee formation of a stable powder with a high yield and minimal amount of moisture content ensuring the reproducibility of the drying technique. In our work, the spray drying technique was used to dry NPs yielding a nano-in-microparticles (NPs-in-MPs) system [26]. In this respect, the yield and moisture content of the prepared SDP were demonstrated. The yield of all SDP ranged from 90.34 ± 5.34% to 93.76 ± 3.12% (Table 3). The use of Man accounted for these high yield values. Although Man has multiple hydroxyl groups, it also has low hygroscopicity. It prevented the adhesiveness of powders to spray drier walls and improved yield [21]. The moisture content of SD/TPP_3_:CS-LPH_o_, as a representative SDP, estimated from the TGA weight loss at temperatures up to 150 °C, was found to be 1.047%. This value was in accordance with those reported in the literature for spray dried formulations [51]. The crystallization of Man on the shell surface reduces the hygroscopicity of the MPs [21].

**Table 3:** Yield, association efficiency and particle size of spray dried TPP cross-linked CS coated LPH_o_

F-code	Yield values (%)	AE^a^ (%)	VMD D[4,3]^b^ (µm)	Span
SD/TPP_1_:CS-LPH_o_	90.34±5.34	96.54±4.23	5.838±0.32	1.507±0.04
SD/TPP_2_:CS-LPH_o_	92.38±3.12^ns^	95.17±3.21^ns^	5.844±0.67^ns^	1.539±0.02
SD/TPP_3_:CS-LPH_o_	93.76±4.51^ns^	97.63±3.28^ns^	4.947±0.89^ns^	2.160±0.38

All results are expressed as mean of 3 determinations ± sd, ^a^ association efficiency. ^b^dry powder: volume mean diameter measured in dry state. Man was used as a drying protectant at 25%w/w. Statistical analysis was carried out using ANOVA followed by Tukey^’^s multiple comparison test to compare SD/TPP_2_:CS-LPH_o_ and SD/TPP_3_:CS-LPH_o_ to SD/TPP_1_:CS-LPH_o_. ^ns^: non-significant (*p *> 0.05).

As for PS, there was a statistically non-significant difference between all values of VMD [4,3] due to the fixation of the feed concentration and technical spray drying parameters (*p* > 0.05) [22]. Interestingly, high association efficiencies (AE) surpassing 94% were attained for all prepared SDP. Only trace amounts of MIR were lost during the spray drying process, as evidenced by the assayed MIR contents, which were extremely close to the theoretical values.

To ensure that the MPs will deaggregate in the GIT after oral administration releasing TPP cross-linked CS-coated LPH (NPs-in-MPs), their recovery was tested in 0.1N HCl and 1 M PBS (pH 7.4). Ten minutes following redispersion in 0.1N HCl, the respective sizes of the obtained NPs for SD/TPP_1_:CS-LPH_o_, SD/TPP_2_:CS-LPH_o_ and SD/TPP_3_:CS-LPH_o_ were 359.9 ± 8.27, 328.1 ± 7.92, and 321.0 ± 3.748 nm, respectively. Recovery indices of 1.08, 1.07, and 1.21 were noted for SD/CS_8_:TPP_1_-LPH_o_, SD/CS_8_:TPP_2_-LPH_o_, and SD/CS_8_:TPP_3_-LPH_o_, respectively approaching 1. This conveys with the literature stating its acceptable range: 0.7–1.3 [24, 52]. Similar results were obtained during recovery of itraconazole nanosuspension from its spray-dried microparticle carrier system [53].

While the PO_4_ groups of TPP are in the ionized form at pH 6–8, the majority of CS amino groups are in the neutral form (NH_2_). As a result, the strength of electrostatic attraction between CS and TPP can be negatively affected, resulting in a heightened swelling [19]. Upon raising pH to 7.4 using 1 M PBS, at the first 10 min, there was a non-significant decrease in PS compared to that in 0.1N HCl (*p* > 0.05). The swelling ratio was calculated by dividing PS at 60 min by that obtained at 10 min. Formula SD/TPP_3_:CS-LPH_o_ exhibited a significant decrease in swelling ratio compared to SD/TPP_1_:CS-LPH_o_, with SD/TPP_2_:CS_-_LPH_o_ having 3.76, 9.24, and 7.42, respectively. The presence of TPP at the highest conc. potentially resisted swelling of particles in PBS (pH 7.4). This was consistent with previous literature. As well stated by Zhou and his colleagues, increasing the cross-linking density of the CS:TPP matrix might have resulted in the lowering of CS swelling ability [19]. By calculating the *R*_index_ at 180 min, all formulae scored values approaching 1 which is optimal. Since, formulae SD/TPP_1_:CS-LPH_o_, SD/TPP_2_:CS_-_LPH_o_, and SD/TPP_3_:CS-LPH_o_ scored 1.15, 1.16, and 1.28 *R*_index_, respectively.

To confirm the recovery of TPP cross-linked CS-coated LPH from the selected SDP and their redispersability, SD/TPP_3_:CS-LPH_o_ was incubated in 0.1N HCl of pH (1.2) at a low stirring rate for 10 min, and the attained dispersion was examined by Fig. 2c, TEM. TEM images (Fig. 2c) show TPP_3_:CS-LPH featuring distinct surfaces with no indications of structural disintegration, suggesting full recovery of the NPs. These images proved that the MPs were readily dispersed in a very short time and sufficiently retained their nano size.

#### In Vitro MIR Release Study

**Fig. 3:**
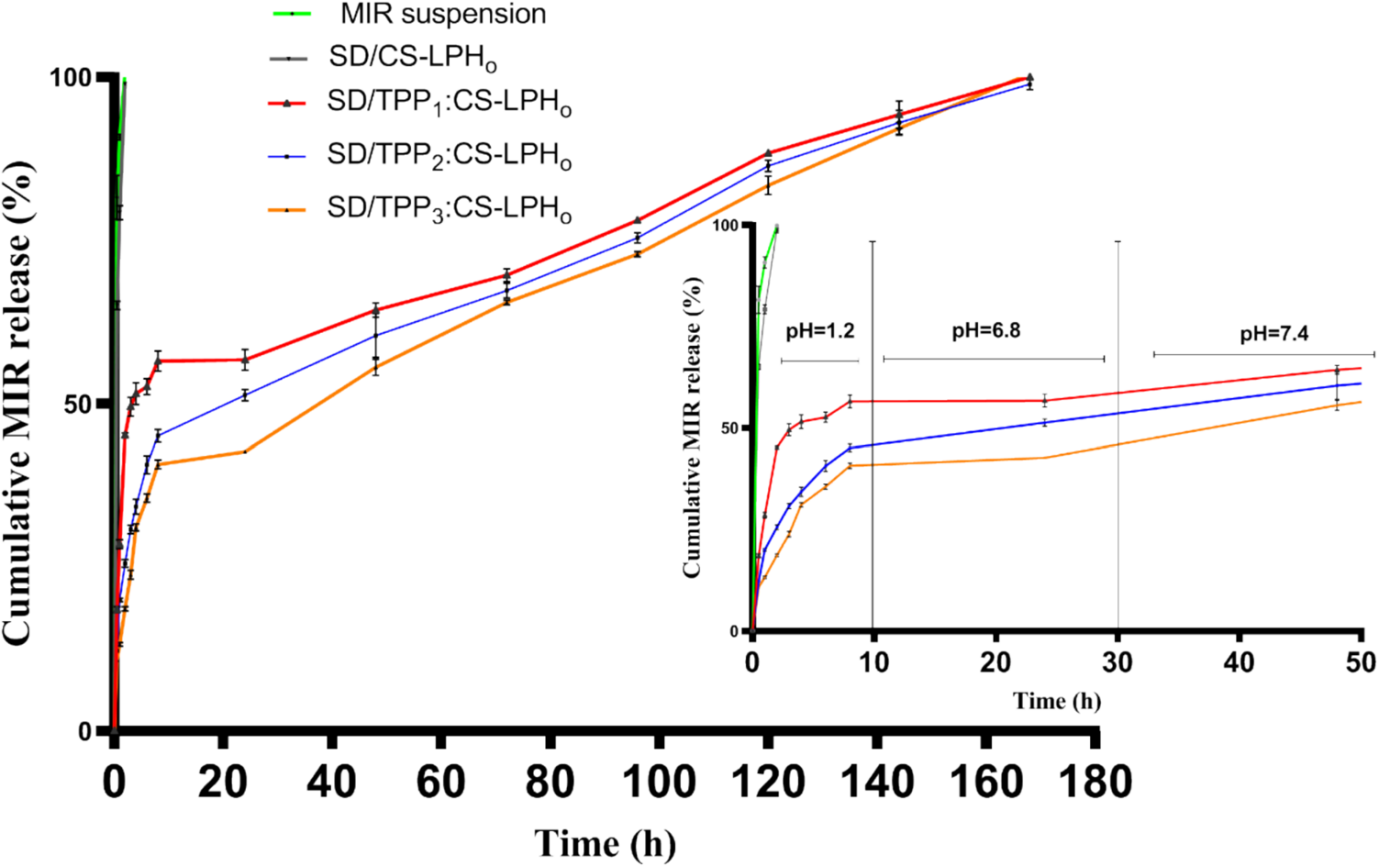
Release profile of MIR from free MIR suspension, SD/CS-LPH_o_, SD/TPP_1_:CS-LPH_o_, SD/TPP_2_:CS-LPH_o_, and SD/TPP_3_:CS-LPH_o_ in different pH at 37 ± 1 °C. Drug concentration in the dialysate was assessed spectrophotometrically. Data points represent mean ± s.d. (*n* = 3)

Figure 3 shows the release profile of MIR from free MIR suspension, SD/CS-LPH_o_, SD/TPP_1_:CS-LPH_o_, SD/TPP_2_:CS-LPH_o_, and SD/TPP_3_:CS-LPH_o_ in different pH at 37 ± 1 °C. As noted in Fig. 3, complete MIR release was achieved within 2 h in HCl from the free drug suspension as well as the SDP of non-cross-linked CS-coated LPH_o_ (SD/CS_8_-LPH_o_). Conversely, the release profile of the TPP cross-linked particles revealed a significant retardation in MIR release (*p* < 0.0001), where MIR release can be arranged in the following descending order: SD/TPP_1_:CS_8_-LPH_o_ > SD/TPP_2_:CS_8_-LPH_o_ > SD/TPP_3_:CS-LPH_o_ with 45.20 ± 0.34, 25.62 ± 0.59, and 18.69 ± 0.30% in 2 h, respectively. This initial release may be due to the drug being loosely adsorbed on the surface or un-entrapped within the particles. Moreover, as TPP conc. increased from 0.066% (SD/TPP_1_:CS_8_-LPH_o_) to 0.2% (SD/TPP_3_:CS-LPH_o_**)**, the release rate of MIR from SDP was found to be inversely related [19].

Upon increasing pH to 6.8, the same trend was observed; as TPP conc. increased from 0.06% to 0.1% and then 0.2% w/v, a decrease in the percent of drug released was noted (52.63 ± 1.22, 40.70 ± 1.37, and 35.65 ± 0.67%, respectively). At pH 7.4, a similar MIR release pattern was noticed at all TPP concentrations. The release rate of MIR from TPP:CS SDP depends on the density of the TPP:CS matrix which increases with increasing amounts of TPP and ultimately slows down the drug release [19]. It is to be noted that MIR release lasted up to 7 days.

Based on the aforementioned findings, the drug release from the formed particles was found to be modulated in a pH-dependent pattern. Since most of CS’s amino groups are protonated at low pH values, strong charge repulsion between molecules can yield an extended conformation. As a consequence, this charged CS has intensified its interaction with TPP molecules, preventing MIR from being released from the cross-linked layer that surrounds LPH_o_ [54]_._ By increasing pH to 6.8 and 7.4, the charge interaction between TPP and CS could be screened by the medium’s ions, resulting in an increase in the percentage of MIR release.

The release data were also quantitatively correlated with zero, first-order, and Higuchi models. The release from SDP of TPP cross-linked CS-coated LPH_o_ followed zero order release. This constant drug release might help in controlling depression which generally follows steady drug levels.

#### Scanning Electron Microscope (SEM)

SEM of the selected SDP of TPP_3_:CS-LPH_o_ shows that the majority of the particles exhibited a wrinkled, corrugated surface (Fig. 2d). The well-established ionic bridging of the particles as a result of surface-bound TPP can explain this wrinkled shape [55]. The increased cross-linking ratio produces a far more asymmetrical shape, which is typical for the materials that form skins and then buckle when cooled [56]. The SDP displays the collapsed vesicular shape, which is typical for CS-based systems resulting from the spray drying technique [57].

#### Fourier Transform-Infrared (FT-IR) Spectroscopy

**Fig. 4:**
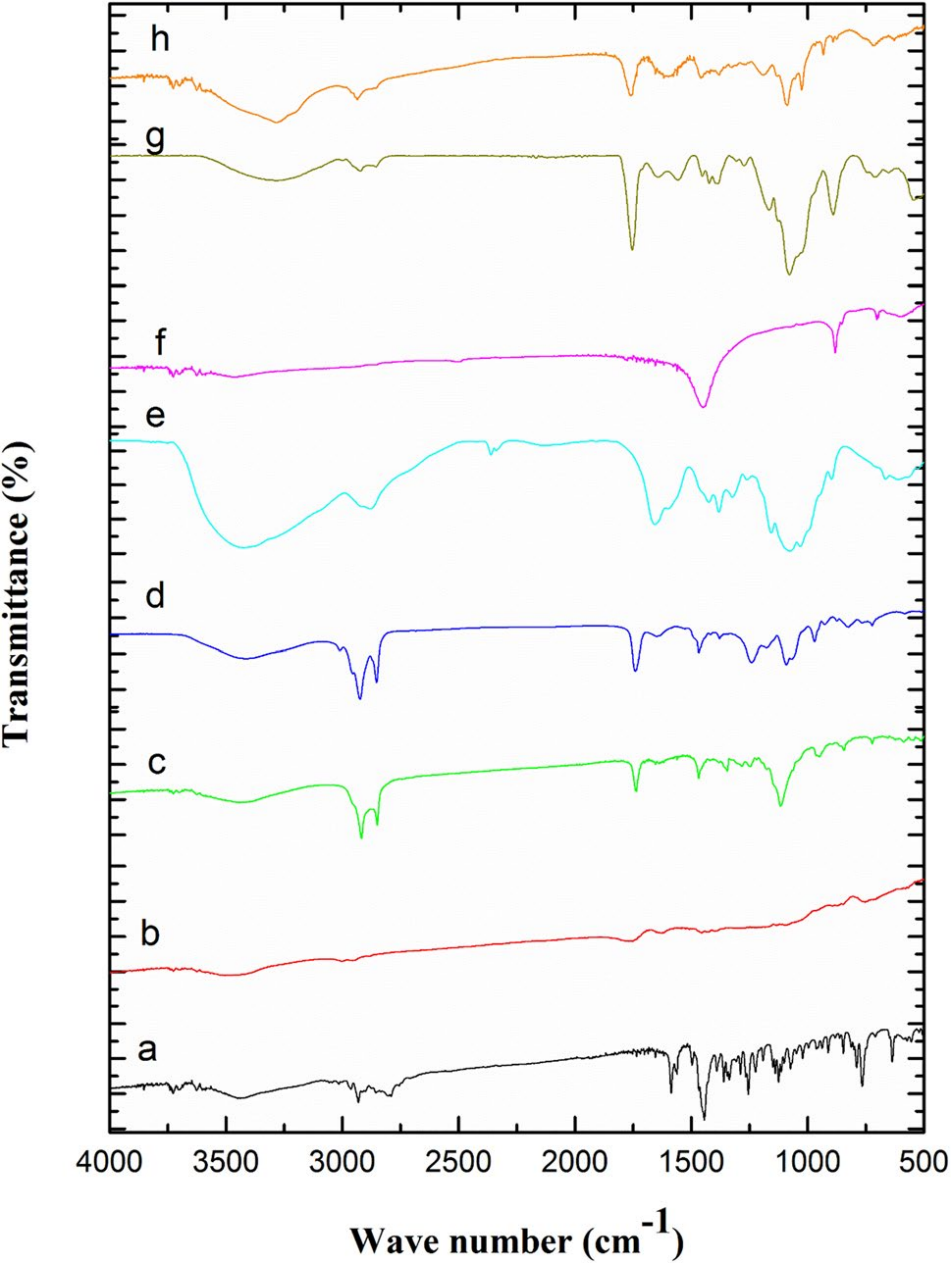
FT-IR spectra of(**a**) MIR, (**b**) PLGA, (**c**) TEF, (**d**) EPC, (**e**) CS, (**f**) TPP, (**g**) plain, and (**h**) medicated SD/TPP_3_:CS-LPH_o_

Figure 4 represents FT-IR spectra of MIR, PLGA, TEF, EPC, CS, TPP, and plain and medicated SD/TPP_3_:CS-LPH_o_. The FT-IR spectrum of plain SD/TPP_3_:CS-LPH_o_ (Fig. 4g) reveals an increase in the peak intensity of the O-H stretching band. This peak appears at 3284 cm^−1^, with a significant shift compared to its position at 3423 cm^−1^ in the CS FT-IR spectrum (Fig. 4e), denoting hydrogen bonding strengthening between phosphate and hydroxyl/amine groups. The cross-linked TPP:CS NPs show the appearance of new peaks at 1272 cm^−1^ that are assigned for P=O asymmetric stretching and at 1070–1130 cm^−1^ which are assigned for P-O-P or P-O stretching [58]. Moreover, an increased intensity was observed in the region of 1020–1070 cm^−1^ due to overlapping between C-O stretching of CS with phosphate peaks. A noticeable shifting of P=O peak was observed from 880 cm^−1^ in the TPP spectrum (Fig. 4f) to 931 cm^−1^ (Fig. 4g), possibly due to the association between the phosphate group of the anionic TPP and the ammonium group of the cationic CS in NPs [45]. An obvious increase in peak intensity at 1758 cm^−1^ was observed, which may be attributed to the overlapping of C=O stretching vibrations of the aliphatic fatty acid chains of both EPC (≈ 1736 cm^−1^) and TEF (similar to triglycerides at ≈ 1740 cm^−1^), as explained in comparative FTIR studies of phospholipids and triglycerides [59].

FT-IR spectrum of medicated SD/TPP_3_:CS-LPH_o_ (Fig. 4h) reveals an increase in the peak intensity of the O-H stretching band that was shifted from 3423 to 3284 cm^−1^ that was well linked to hydrogen bonding [60]. A noticeable decrease in the intensity of the C=O stretching of the aliphatic fatty acid chains of EPC at 1758 cm^−1^ was observed which suggests the existence of electrostatic interaction with the cationic quaternary ammonium group of MIR besides the contribution of hydrophobic interactions in the developed NPs [61]. The amide I band of CS was dramatically shifted from ~1550–1655 cm^−1^ to 1617 cm^−1^ and broadened, possibly due to the aromatic skeletal vibration of MIR at 1500–1600 cm^−1^, altering chitosan’s amine environment [62]. A significant decrease in the intensity of phosphate groups at 1070–1130 cm^−1^ and at 931 cm^−1^ which is assigned for P-O-P or P-O stretching might be due to the incorporation of MIR that partially shields phosphate groups [63].

#### Differential Scanning Calorimetry (DSC)

**Fig. 5:**
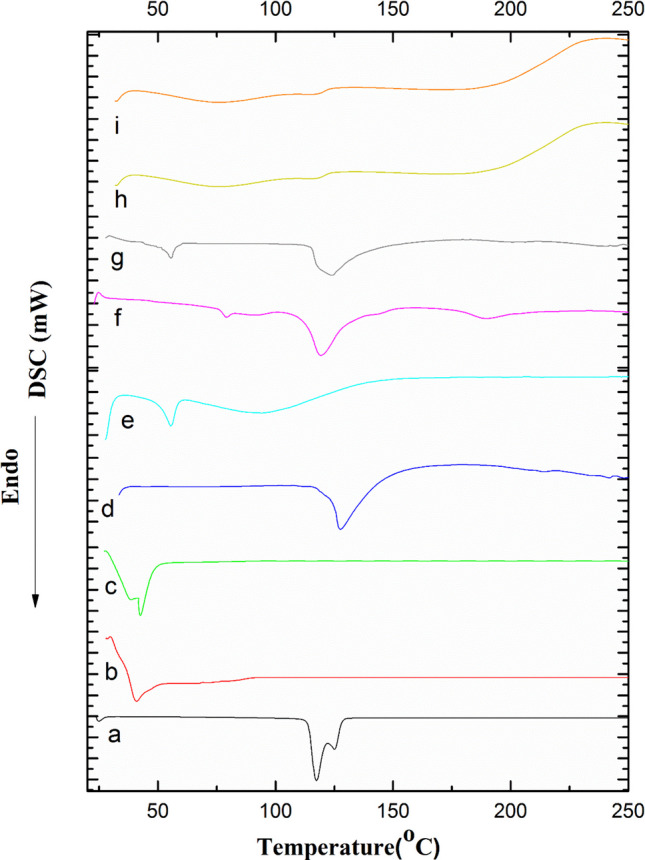
DSC thermograms of(**a**) MIR, (**b**) PLGA, (**c**) TEF, (**d**) EPC, (**e**) CS, (**f**) TPP, (**g**) 1:1:1:1:1:1 physical mixture of MIR:PLGA:TEF:EPC:CS:TPP, and (h) plain and (i) medicated SD/TPP_3_:CS-LPH_o_

Figure 5 shows DSC thermograms of MIR, PLGA, TEF, EPC, CS, and TPP and the physical mixture of 1:1:1:1:1 of MIR:PLGA:EPC:TEF:TPP:CS, and plain and medicated SD/TPP_3_:CS-LPH_o_. Thermal analysis was employed to assess the crystalline or amorphous behavior of MIR in SD/TPP:CS-LPHo and to probe any possible variations in the physical attributes of the individual components of the proposed system. The DSC thermogram of the MIR:PLGA:EPC:TEF:TPP:CS physical mixture was a simple superposition of peaks of the individual components as shown in Fig. 5g. The DSC thermograms of both the plain and drug-loaded formula SD/TPP_3_:CS-LPH_o_ (Fig. 5h, i) were almost superimposed. Moreover, the intensity of the characteristic TPP peak at 189 °C (Fig. 5f) was significantly decreased, confirming the effective cross-linking between CS and TPP [45]. Furthermore, this thermogram reveals the absence of endothermic peaks of MIR indicating its presence as molecular dispersion inside the attained matrix.

#### X-ray Powder Diffraction (XRPD)

**Fig. 6:**
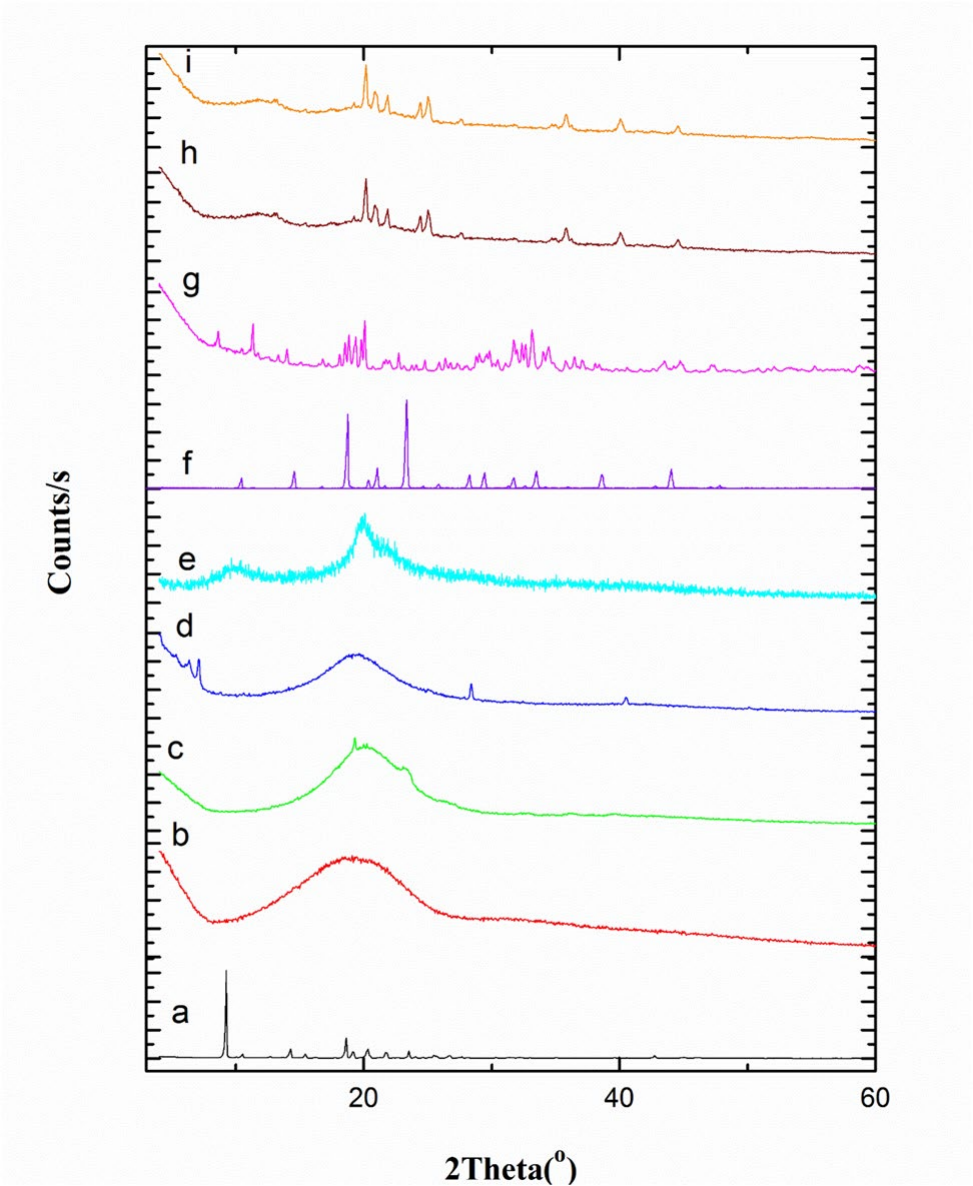
X-ray diffractograms of(**a**) MIR, (**b**) PLGA, (**c**) TEF, (**d**) EPC, (**e**) CS, (**f**) Man, (**g**) TPP, and (**b**) plain and (**i**) medicated SD/TPP_3_:CS-LPH_o_

Figure 6 illustrates X-ray diffractograms of MIR, PLGA, TEF, EPC, CS, Man, TPP, and plain and medicated SD/TPP_3_:CS-LPH_o_. The diffractogram of MIR indicates the highly crystalline nature of MIR with prevalent and strong diffraction peaks appearing across the range of 2θ values of 9.5, 14.5, 19.0, and 20.7° (Fig. 6a). The amorphous nature of PLGA, TEF, and EPC is evidenced in Fig. 6b–d, respectively. The diffractogram of CS (Fig. 6e) represents the typical fingerprints of the semi-crystalline polymer [64]. The Man diffractogram (Fig. 6f) reveals crystalline peaks at 10.56° and 14.71°, pinpointing the existence of the beta polymorph, in addition to other lower intensity peaks at 19, 24, 40, and 45° [27]. The XRD diffractogram of TPP (Fig. 6g) exhibits several prominent peaks ranging from 11.3 to 74°; the most noticeable ones were noticed at 19.05, 19.77, 33.60, and 34.49° [55].

The Plain SD/TPP_3_:CS-LPH_o_ diffractogram (Fig. 6h) reveals the presence of the two CS peaks at very low intensity, indicating the effective cross-linking between CS and TPP with Man peaks of *α* and *β* polymorphs that result after SD. These Man peaks were noted in previous studies after SD of Man. It is evident that even though the SD process can produce amorphous powders, Man opposed complete powder amorphization [27]. MIR-SD/TPP_3_:CS-LPH_o_ diffractogram (Fig. 6i) was similar to that of the plain formula with none of the drug peaks. Its distinct crystal peaks vanished as they overlapped with the polymer noise, signifying full MIR amorphization.

#### Stability Study

One of the major problems encountered following the spray drying is the agglomeration and fusion of the particles during storage. Accordingly, a stability study was conducted on the selected SD/TPP_3_:CS-LPH_**o**_ and stored in a silica desiccator for a period of 6 months, by physical observation of the powder and monitoring changes in the PS and AE%. As shown in Table S3, no significant change was noticed in the VMD (D[4,3]) of SDP after the first and third month, measured by mastersizer confirming the stability of the selected MPs under ambient conditions (*p* > 0.05). After 6 months, a significant increase in PS value was demonstrated; however, it still did not affect the oral administration. As for AE%, non-significant differences were noticed relative to the freshly prepared SDP during the whole 6 months (*p* > 0.05). Moreover, no visible agglomeration was observed reflecting the suitability of Man as a drying agent.

### In Vivo* Study*

#### Pharmacokinetic Study

**Fig. 7:**
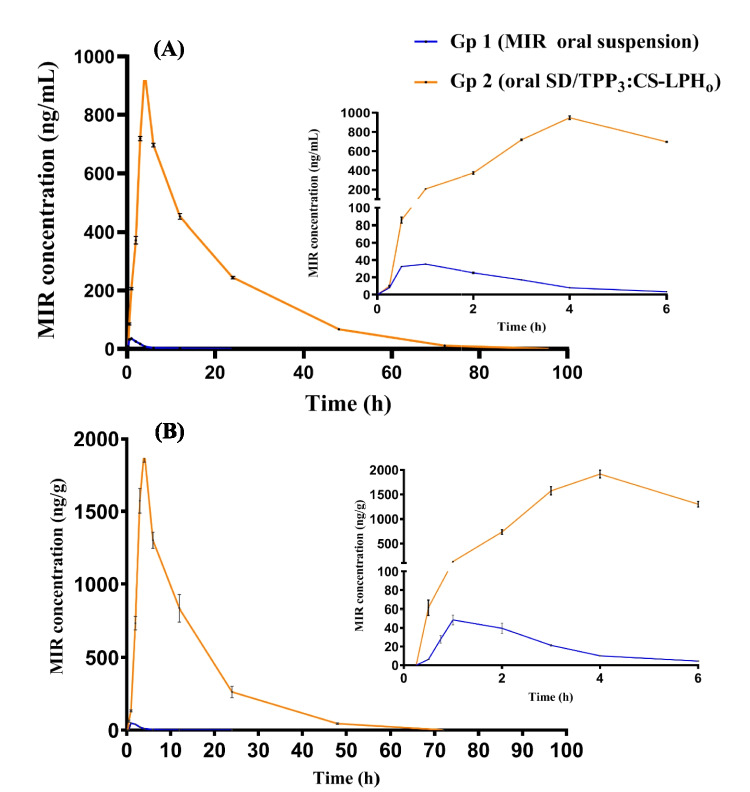
Mean MIR concentrations in the pharmacokinetic study in **A** plasma and **B** brain homogenate concentrations versus time profiles obtained following administration of MIR loaded formula. *Gp1 received oral MIR suspension and Gp 2 received oral SD/TPP_3_:CS-LPH_o_. The doses of Gp 1 and Gp 2 were normalized to 1 mg/kg in male Swiss mice (*n* = 4)

Figure 7 shows the mean MIR concentrations in plasma and brain homogenate (BH) post oral administration of MIR suspension (gp 1) and SD/TPP_3_:CS-LPH_o_ (gp 2) following dose normalization to 1 mg/kg. The concentration of MIR in plasma was detected until 12 and 72 h for gps 1 and 2 (SD/TPP_3_:CS-LPH_o_) respectively. The highest plasma MIR concentration was achieved after 1 and 4 h for gp 1 (oral MIR suspension) and gp 2 (oral SD/TPP_3_:CS-LPH_o_), respectively. Table 4 shows MIR plasma and BH pharmacokinetic parameters obtained following administration of the MIR-loaded formula to mice. A significantly lower *C*_max, plasma_ was obtained following oral administration of MIR suspension (gp 1) compared to the SD formula (gp 2) with respective values of 35.39 ± 0.36 ng/mL and 948.35 ± 19.24 ng/mL. Moreover, administration of the SD formula significantly extended *t*_max, plasma_ compared to the free drug where their respective *t*_max, plasma_ was 4 and 1 h (*p* < 0.05), respectively. This can be rationalized by the presence of TPP cross-linked CS coating LPH which was more stable in the stomach and delayed the drug release in the GI fluids [65]. A similar observation was reported following oral administration of CS-coated liposomes loaded with berberine to rabbits with a recorded delay in *t*_max, plasma_ of up to 4 h [25]. Indeed, the potential of CS to open tight junctions, expediting paracellular transport is well-documented [65]. Similarly, a significantly higher AUC_0-t, plasma_ was obtained following orally administered gp 2 (oral SD/TPP_3_:CS-LPH_o_) compared to gp 1 with respective values of 15,714.46 ± 195.74 and 113.09 ± 3.43 ng/mL*h. Gp 2 achieved a 139-fold increase in AUC_0-t, plasma_ (*t* was the last measurable concentration) compared to gp 1. Noticeably, higher values of *t*_1/2, plasma_ for gp 2 with 11.19 ± 0.14 h compared to gp 1 with 2.70 ± 0.27 h confirmed the remarkable prolongation of drug release. This observation was consistent with the aforementioned results of in vitro release; the release of the oral formula was more extended over a period of 7 days. The pegylation effect of the proposed pegylated LPHNPs can also be verified from the average time that the drug stays in the body; the MRT_plasma_ significantly higher value of MRT_plasma_ of gp 2 was attained with 17.82 ± 0.18 h compared to gp 1 which showed an MRT_plasma_ value of 3.50 ± 0.11 h. The pegylation effect was also evidenced by achieving a significantly lower value of the apparent volume of distribution (Vz/F) in plasma in the case of gp 2 with 0.10 ± 0.01 L/kg compared to gp 1 with 32.84 ± 3.81 L/kg.

**Table 4:** MIR plasma pharmacokinetic and brain homogenate parameters obtained following administration of MIR-loaded formulae to mice

	Gp 1		Gp 2	
Parameter	Plasma	Brain homogenate	Plasma	Brain homogenate
Cmax (ng/mL)	35.39±0.36	48.58±5.21	948.35±19.24	1915.23±78.76
Tmax (h)	1±0	1±0	4 ±0	4 ±0
t_1/2_(h)	2.70±0.27	1.40±0.15	11.19±0.14	6.60±0.10
AUC_0-t_^a^ (ng.h/mL)	113.09±3.43	119.25±17.63	15714.46±195.74	23837.73±1999.26
AUC_0-∞_ (ng.h/mL)	119.26±3.94	129.07±6.98	15894.95±208.24	23846.75±1998.98
MRT	3.50±0.11	2.70±0.21	17.82±0.18	12.77±0.30
Vz/F (L/kg)^*^	32.84±3.81	16.44±2.82	0.10±0.01	0.41±0.04
CL/F (L/h)**	0.21±0.01	0.20±0.01	0.01±0.00	0.001±0.00

All results are expressed as mean ± standard error. ^a^ t is the last measurable concentration. *Vz/F: apparent volume of distribution and **CL/F: apparent clearance after oral administration. Pl: plasma and BH: brain homogenate. Gp 1: received oral MIR suspension and gp 2: received oral SD/TPP_3_:CS-LPH_o_, where data was calculated after normalizing dose of Gps 1 and 2 data to 1mg/kg, in Swiss mice (n=4).

As shown in Fig. 7, MIR could be detected in BH after oral administration of MIR suspension (gp 1) after 30 min and *C*_max brain_ was achieved at 1 h (Table 4). A significantly higher (*p* < 0.05) value of *t*_max brain_ of gp 2 (oral formula) (4 h) compared to gp 1 (oral MIR suspension) after 1 h was noticed, indicating the retardation of drug absorption from the oral formula from the gastrointestinal tract (GIT), as explained before in plasma PKs. The concentration of MIR in BH was detected until 6 and 48 h for gps 1 and 2 (oral MIR suspension and SD/TPP_3_:CS-LPH_o_), respectively.

A 39-fold increase in *C*_max brain_ was seen with the SD formula (SD/TPP_3_:CS-LPH_o_) (gp 2) compared to oral MIR suspension (gp 1) (*p* < 0.05) with respective values of 1915.23 ± 78.76 and 48.58 ± 5.21 ng/g, pinpointing a dramatic boost in MIR brain concentration, as shown in Table 4. The same trend was also noticed regarding AUC values where the oral SD formula possessed a statistically relevant higher AUC_0-t brain_ value in comparison to the free drug suspension (*p* < 0.05) with a 199-fold increase. It exhibited an extended *t*_1/2_ equivalent to 4.7 times that of free MIR suspension (*p* < 0.05), indicating the efficiency of pegylation in prolonging circulation time.

The incorporation of TEF with its PEG groups in the LPHNPs could have decreased protein adsorption and slowed down the LPHNPs clearance improving blood circulation time; as a consequence, pegylated LPHNPs could accumulate more efficiently in the brain [66]. Referring to literature, a more than 40-fold increase in *C*_max_ was reported after oral administration of nanoemulsions curcumin (NEC) containing PEG in mice [67].

Formulating MIR in LPHNPs with subsequent spray drying utilizing chitosan-TPP self-assembly could overcome challenges of oral delivery of drug suspension. Since, the mucoadhesive properties of chitosan greatly improved drug absorption by coating the LPHNPs and facilitated its penetration through the gastrointestinal wall [68]. The PEG chains’ ability to sterically impede plasma protein binding contributes towards better stability of the proposed LPH. Moreover, the pharmacokinetics are eventually improved in terms of enhanced biodistribution and retention at the intended site of action [69].

#### Pharmacodynamic Study

**Fig. 8:**
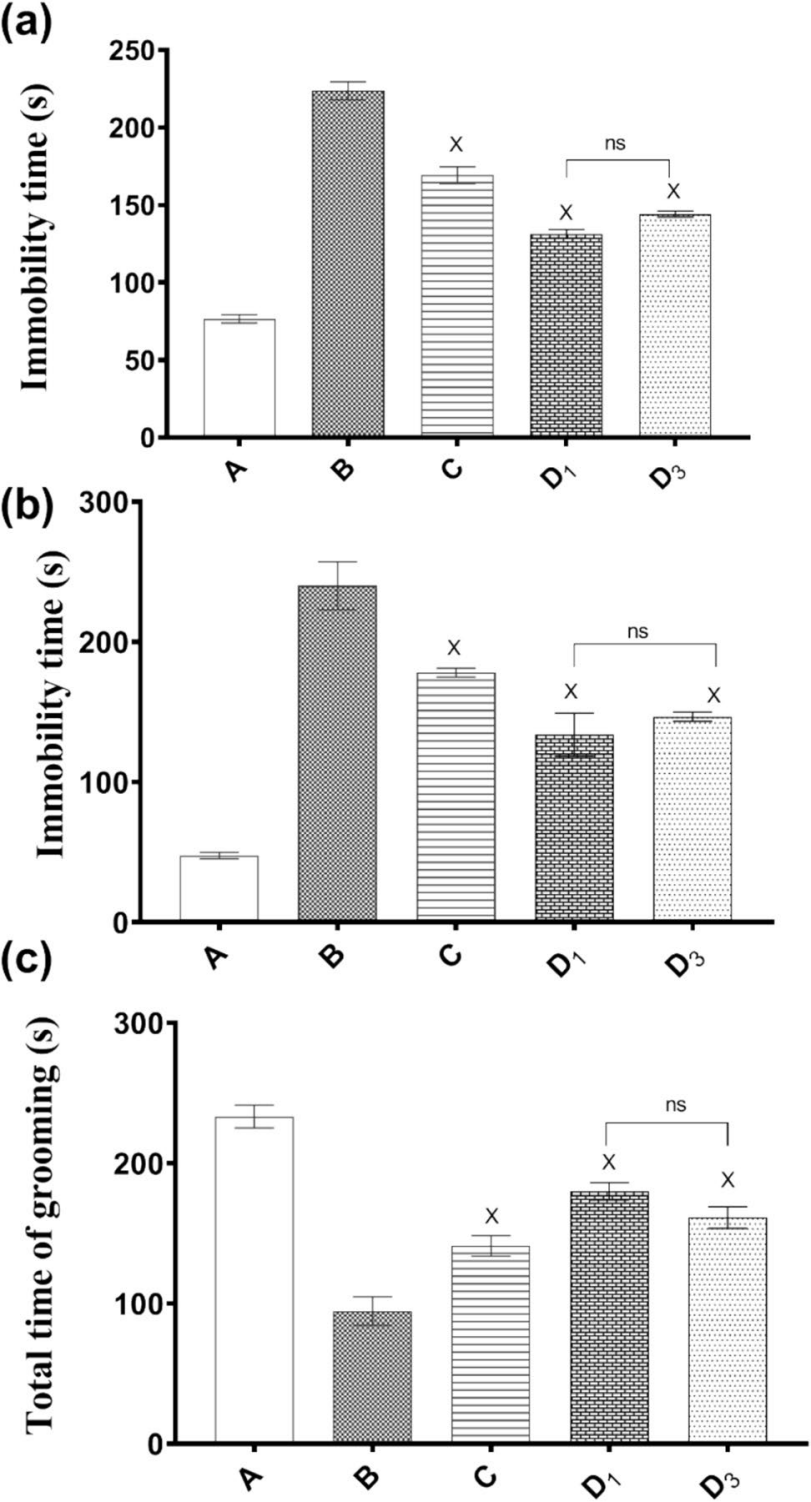
Effect of treatment with selected formula on immobility time in the FST (**a**), TST (**b**), and the time spent grooming in the splash test (**c**) in mice submitted to the CUMS procedure. Gp A, non-stressed (normal mice); gp B, stressed, non-treated (CUMS); gp C, stressed, oral MIR suspension daily; gp D_1_, stressed, oral (SD/TPP_3_:CS-LPH_o_) daily; and gp D_3_, stressed, oral (SD/TPP_3_:CS-LPH_o_) every 3 days. Results are expressed as means ± S.E.M. (*n* = 5). Statistical analysis was performed for each group compared to (A) non-stressed and (B) stressed, non-treated groups using one-way ANOVA followed by Tukey’s test for multiple comparisons. X indicates *p* < 0.0001 as compared with B. ns, non-significant difference (*p* > 0.05)

To ascertain the aptness of the chosen orally administered MIR-loaded SDP for the treatment of depression, a pharmacodynamic study was carried out. Figure 8 shows the effect of treatment with the selected formula on the immobility time in the FST, TST, and ST in mice submitted to the CUMS procedure.

#### Behavioral Tests

The behavioral despair test, or FST, is based on how a rodent responds to the impedance of drowning; the test’s findings have been used to gauge a subject’s vulnerability to negative behavior. The CUMS paradigm in stressed non-treated mice (gp B) revealed a statistically significant ~threefold increase in the immobility time compared to normal, non-stressed mice of gp A (*p* < 0.0001) (Fig. 8a). All stressed mice treated with MIR demonstrated significant decrements in immobility time (*p* < 0.0001) relative to the stressed non-treated gp B. Comparing MIR oral formulae revealed that the selected SD/TPP_3_:CS-LPH_o_ performed better relative to oral MIR suspension as manifested by the significantly shorter immobility time (*p* < 0.0001), whatever its dosing regimens. Comparing both *D*_1_ and *D*_3_ gps, orally treated with SD/TPP_3_:CS-LPH_o_ either daily or every 3 days, respectively, showed non-significantly different values (*p* > 0.05). These results could verify the prolonged release of MIR offered by the oral formula allowing for their 3-day dosing regimen.

TST is a widely used behavioral test reflecting the depression state in mice, causing a prolongation in the immobility time in CUMS mice. Fruitfully, the two dosing regimens showed non-significant differences, indicating efficient MIR release prolongation (*D*_1_–*D*_3_) at *p* > 0.05 (Fig. 8b). This release prolongation offered by the selected oral SD formula yielded better performance relative to oral MIR suspension as manifested by its significantly shorter immobility time (*p* < 0.0001), whatever its dosing regimen.

ST was conducted to evaluate the depressive-like behavior of mice. Grooming behavior is a reflective measure of depressive symptoms in a reverse pattern. Similar to that observed in TST, the two dosing regimens showed non-significant differences in grooming time (*D*_1_–*D*_3_) at *p* > 0.05 (Fig. 8c) further confirming MIR release prolongation.

#### Biochemical Markers Change

The neurotrophin family member, BDNF, is crucial for the pathophysiology of depression and responds to the antidepressants, being capable of mediating their clinical effects [70]. Being a factor supporting neuronal survival, BDNF levels have been reported to decrease in both the prefrontal cortex (PFC) and hippocampus associated with reduced neurogenesis in multiple studies that used the CUMS protocol [30]. Antidepressant medication can raise BDNF levels and promote its neurogenesis effect [30]. Figure 10 shows the effect of treatment with the selected formula on BDNF and 5HT levels in CUMS-exposed mice.

**Fig. 9:**
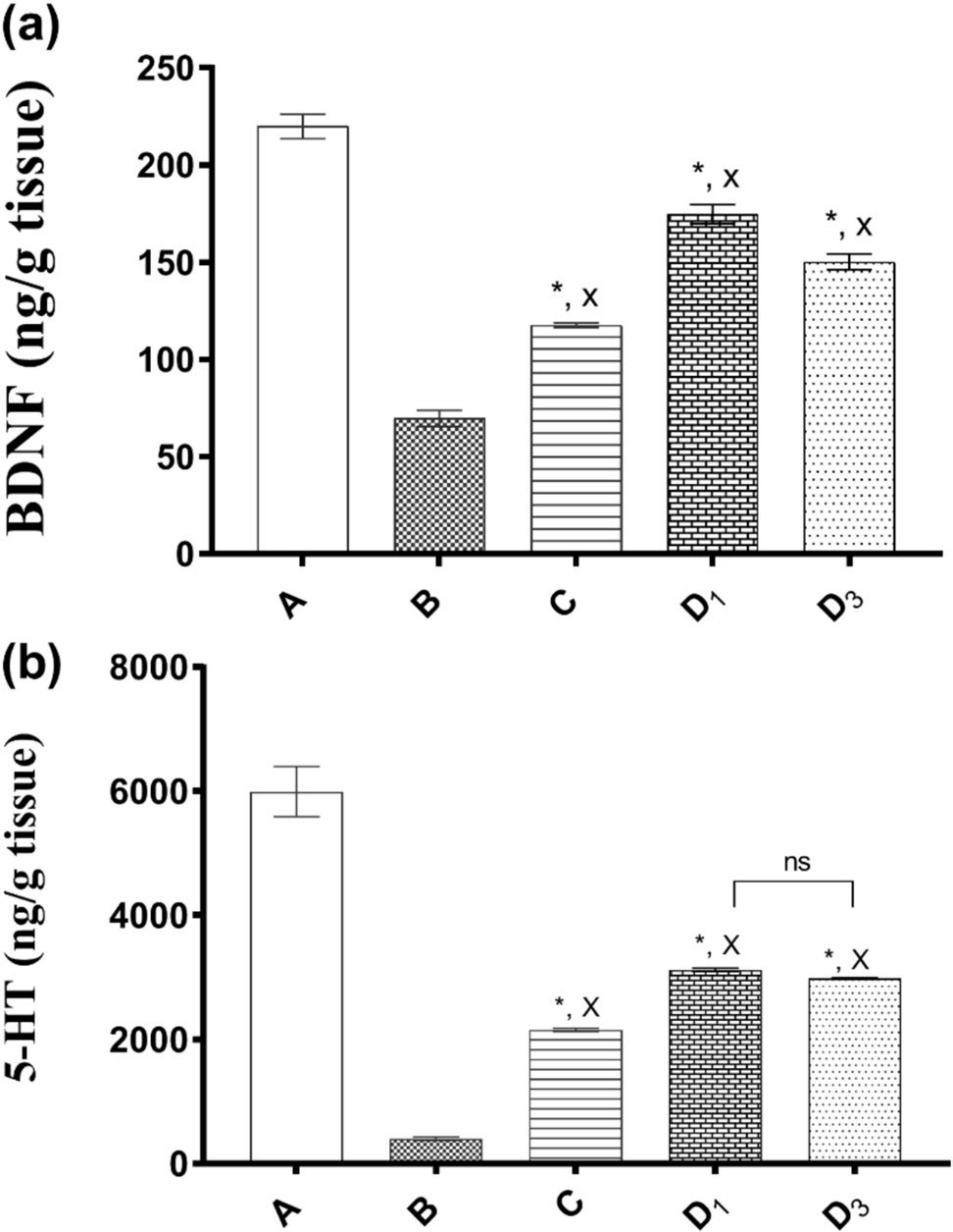
Effect of treatment with selected formula on **a** BDNF and **b **5-HT levels in CUMS-exposed mice. Gp A, non-stressed (normal mice); gp B, stressed, non-treated (CUMS); gp C, stressed, oral MIR suspension daily; gp *D*_1_, stressed, oral (SD/TPP_3_:CS-LPH_o_) daily; and gp *D*_3_, stressed, oral (SD/TPP_3_:CS-LPH_o_) every 3 days. Results are expressed as means ± S.E.M. (*n* = 3). Statistical analysis was conducted for each group compared to (A) non-stressed and (B) stressed, non-treated groups using one-way ANOVA followed by Tukey’s test for multiple comparisons. Asterisk (*) and X indicate *p* < 0.0001 as compared with B and A, respectively. ns, non-significant difference (*p* > 0.05)

Herein, BDNF level in normal mice was found to decrease by inducing depression by CUMS protocol as shown in Fig. 9a, further confirming the successful induction of depression. The CUMS paradigm in gp B showed a significant decrease in BDNF level by 68.3% in comparison with the non-stressed mice (gp A) (respective values were 69.7 ± 7.34 and 219.9 ± 10.83 ng/g protein) at *p* < 0.0001.

Assessing the conventional MIR dosage form (oral MIR suspension) revealed its lowest antidepressant effectiveness when given daily (gp C) showing a significantly higher value of BDNF level by 1.68-fold, in comparison with the CUMS paradigm (gp B). The reverse was true in comparison with the non-stressed mice (gp A) showing a 0.53-fold decrease in BDNF level at *p* < 0.0001. These results could indicate its inability to achieve complete restoration to the normal level of (gp A), highlighting the possibility of extending the duration of treatment to more than 7 days in future studies.

As for orally administered groups with (SD/TPP_3_:CS-LPH_o_), gps *D*_1_ and *D*_3_, either daily regimen or every 3 days, respectively, showed significantly higher values in the level of BDNF by 2.51- and 2.35-fold, respectively, at *p* < 0.0001 compared to the CUMS paradigm. Thus, their appropriate anti-depressant potentials were verified; yet, complete curness could not be achieved, attaining significantly lower marker levels at *p* < 0.001 compared to normal mice non-stressed (gp A).

It should be noted that orally administered gps *D*_1_ and *D*_3_, whatever the dosage regimen, showed significantly higher values of BDNF levels by 1.49- and 1.40-fold, respectively, relative to that of stressed mice treated using daily oral MIR suspension (gp C) at *p* < 0.0001. Such findings indicated their superiority over oral MIR suspension.

It is worthmentioning that MIR suffers from different limitations when given orally in free form; MIR undergoes first-pass metabolism after being rapidly absorbed from the GIT. It suffers from 80% plasma protein binding; thus, designing it in LPHNPs yields better performance and bioavailability, translated by excellent pharmacodynamic response.

Consequently, oral formula can be administered every 3 days not on a regular basis, as comparing both gps *D*_1_ and *D*_3_ showed a non-significant difference (*p* > 0.05). In line with previous studies, formula (SD/TPP_3_:CS-LPH_o_) for oral treatment ameliorated CUMS-induced decreased neurogenesis and BDNF level.

The current study evaluated PFC and hippocampal 5-HT because it is considerably involved in the depression modulation [30]. As obvious in Fig. 9b, the level of 5-HT was significantly depleted in stressed mice (~15-fold decrease in 5-HT level) relative to that of the non-stressed mice (gp A) at *p* < 0.0001, which was consistent with earlier works, confirming the incidence of depression [30]. The same pattern was observed with the BDNF level as mentioned above.

Daily treatment of stressed mice with oral MIR suspension (gp C) resulted in significantly higher values of 5-HT level by 5.38-fold, in comparison with the CUMS paradigm (gp B) at *p* < 0.0001. However, approaching normal 5-HT levels of non-stressed mice (gp A) was not accomplished as gp C showed a significantly lower value of 5-HT level by 0.36-fold, relative to that of the non-stressed mice (gp A) at *p* < 0.0001.

Both gps *D*_1_ and *D*_3_, treated stressed mice using orally administered formula (SD/TPP_3_:CS-LPH_o_) either in daily regimen or every 3 days, showed significantly higher values of 5-HT by 7.80- and 7.46-fold, respectively, at *p* < 0.0001 compared to CUMS paradigm, highlighting its anti-depressant efficacy.

#### Brain Histopathological Examination

**Fig. 10:**
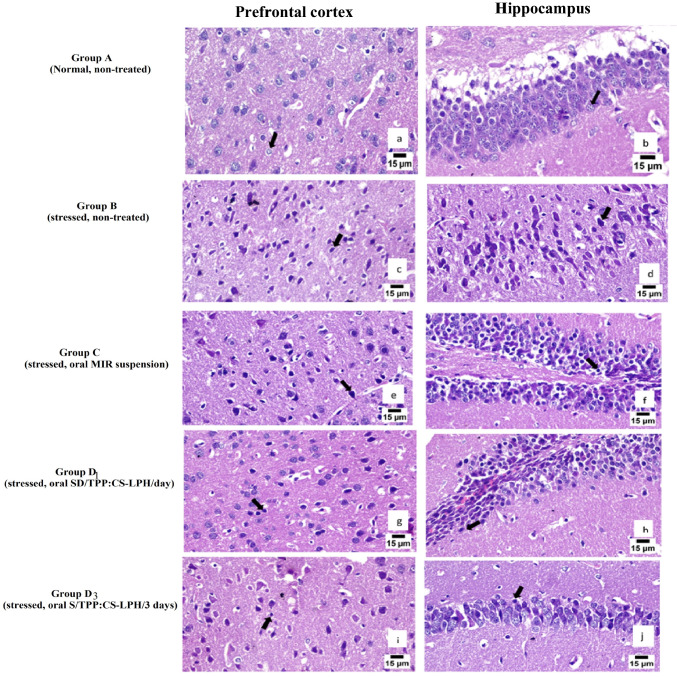
Histopathological brain sections of prefrontal cortex and hippocampus of the studied groups. Group A, normal, non-treated; group B, stressed, non-treated; group C, stressed, treated daily with oral MIR suspension; group D_1_, stressed, treated daily with oral SD/TPP_3_:CS-LPH; and group D_3_, stressed, treated every 3 days with oral SD/TPP_3_:CS-LPH. Composition of SD/TPP_3_:CS-LPH: 1%w/v PLGA concentration, 15.39%w/w lipid concentration, 5%w/w MIR concentration, 0.2%w/v CS, 0.2%w/v TPP, and 0.077%w/v Man

Indeed, chronic stress causes several structural changes in depressed brains in the form of a decrease in hippocampal volume that leads to CUMS-induced anhedonia [71, 72]. Moreover, exposure to CUMS lowered the expression of BDNF and other neurotrophins, causing shrinkage of neuronal dendrites of the hippocampus and disruption of the prefrontal cortex [73]. From brain-targeted medication perspectives, the efficacy of treatment with antidepressants can be assessed by examining the neuronal changes in the hippocampus and prefrontal cortex [30]. Figure 10 shows the histopathological brain sections of the prefrontal cortex and hippocampus of the study groups.

In the normal mice gp A (Fig. 10a, b), both PFC and hippocampus showed round neurons with well-organized intact structures [73]. Compared with the normal mice gp, the PFC of the CUMS gp showed diffused gliosis with necrotic neurons. The CUMS gp’s hippocampal neurons displayed karyopyknosis and were deeply stained showing irregular, spindle-shaped polygons (Fig. 10c, d). They were loosely arranged and extensively degenerated [73].

Figure 10e and f demonstrate the histopathologic alterations in the nerve cells in PFC and hippocampus of gp C (stressed, treated with oral MIR suspension). An increased number of dark degenerated neurons with gliosis in the PFC was noticed. The hippocampal neurons exhibited irregular morphology and were loosely arranged.

Furthermore, marked improvement was noticed on observing Fig. 10g and h that show the histopathologic changes in the neurons of PFC and hippocampus of gp *D*_1_ (stressed and administered daily the oral formula (SD/TPP_3_:CS-LPH_o_). In the mouse hippocampal regions, the majority of neurons were robust in structure with a small percentage displaying karyopyknosis, indicating fewer degenerated neurons.

Meanwhile, fewer dark degenerations of pyramidal cells in the hippocampus were observed in gp *D*_3_ that was stressed and administered the oral formula (SD/TPP_3_:CS-LPH_o_) every 3 days starting from day 7 to day 14, i.e., received three doses only to the end of the study (Fig. 10i, j). Most of the neurons were intact in the PFC.

## Conclusion

In this study, MIR was successfully loaded in LPH and integrated with TPP-crosslinked CS coat using spray drying in an attempt to achieve optimal brain-directed oral delivery. MIR-loaded LPH in this study resulted in formulations with low nanoscale particle sizes, spherical shape and high EE%. The TPP:CS-LPH showed slightly smaller particle size relative to non-crosslinked uncoated ones and a positive zeta potential. In vitro drug release studies showed that MIR was slowly released from TPP-crosslinked formulations compared to uncoated LPH. Oral delivery of SD/TPP:CS-LPH exhibited better pharmacokinetic/pharmacodynamic parameters over oral MIR suspension. This tailored oral platform serves as a possible venue for brain-directed delivery. The main anticipated consequences are long residence time, boosted bioavailability, optimal therapeutic efficacy, and diversified treatment alternatives.

## Supplementary Information

Below is the link to the electronic supplementary material.

ESM 1 (DOCX 599 KB)

## Data Availability

Data can be provided by the authors upon request.
