# Supplementary material for: Cross-linked Chitosan-Based Shell with Mirtazapine Lipid Polymer Hybrid Core as Integrated Spray-Dried Bionanocomposites for Boosted Brain-Directed Oral Delivery
Source: Mol Neurobiol. 2025 Dec 8;63(1):269. doi: 10.1007/s12035-025-05403-5 (PMC12686112; doi:10.1007/s12035-025-05403-5)
Supplement: Supplementary file 1 — (DOCX 599 KB) [file 12035_2025_5403_MOESM1_ESM.docx]

**Cross-linked chitosan-based shell with mirtazapine lipid polymer hybrid core as integrated spray dried bionanocomposites for boosted brain-directed oral delivery**

**Molecular neurobiology**

**Dalia M. Elbehairy, Enas Elmowafy*, Rihab Osman, Omaima A Sammour**

*Department of Pharmaceutics and Industrial Pharmacy, Faculty of Pharmacy, AinShams University, Cairo, Egypt,Monazzamet Elwehda Elafrikeya Street, Abbaseyya, Cairo, Egypt, P.O.B. 11566*

*** Corresponding author:**

**Enas Elmowafy**

*Department of Pharmaceutics and Industrial Pharmacy, Faculty of Pharmacy, Ain Shams University, Cairo, Egypt,Monazzamet Elwehda Elafrikeya Street, Abbaseyya, Cairo, Egypt, 11566.*

Tel.:(+202)01140380412

Email: [Enasmostafa@pharma.asu.edu.eg](mailto:Enasmostafa@pharma.asu.edu.eg)

**Table (S1): Significance of CMAs effects and interactions on PS, EE and ζ in MIR-LPH.**

| Factor | CQA | Sum of squares | Df | Mean square | *F* value | *P* value  Prob>*F* | Significance (*P*<0.05) |
| --- | --- | --- | --- | --- | --- | --- | --- |
| Model | PS | 6.282E-003 | 9 | 6.980E-004 | 276.64 | <0.0001 | Significant |
|  | EE | 2.067E-003 | 9 | 2.297E-004 | 79.72 | <0.0001 | Significant |
|  | ζ | 417.33 | 9 | 46.37 | 72.92 | < 0.0001 | Significant |
| A (PLGA) | PS | 5.856E-003 | 1 | 5.856E-003 | 2321.13 | < 0.0001 | Significant |
|  | EE | 2.473E-004 | 1 | 2.473E-004 | 85.82 | < 0.0001 | Significant |
|  | ζ | 236.42 | 1 | 236.42 | 371.78 | < 0.0001 | Significant |
| B (L) | PS | 4.368E-005 | 1 | 4.368E-005 | 17.31 | 0.0042 | Significant |
|  | EE | 8.455E-005 | 1 | 8.455E-005 | 29.35 | 0.0010 | Significant |
|  | ζ | 14.82 | 1 | 14.82 | 23.31 | 0.0019 | Significant |
| C (MIR) | PS | 5.468E-005 | 1 | 5.468E-005 | 21.67 | 0.0023 | Significant |
|  | EE | 1.159E-003 | 1 | 1.159E-003 | 402.20 | < 0.0001 | Significant |
|  | ζ | 32.56 | 1 | 32.56 | 51.20 | 0.0002 | Significant |
| AB | PS | 1.950E-005 | 1 | 1.950E-005 | 7.73 | 0.0273 | Significant |
|  | EE | 1.473E-008 | 1 | 1.473E-008 | 5.111E-003 | 0.9450 | Non-significant |
|  | ζ | 0.093 | 1 | 0.093 | 0.15 | 0.7135 | Non-significant |
| AC | PS | 6.891E-006 | 1 | 6.891E-006 | 2.73 | 0.1424 | Non-significant |
|  | EE | 2.616E-004 | 1 | 2.616E-004 | 90.81 | < 0.0001 | Significant |
|  | ζ | 7.73 | 1 | 7.73 | 12.15 | 0.0102 | Significant |
| BC | PS | 4.872E-007 | 1 | 4.872E-007 | 0.19 | 0.6736 | Non-significant |
|  | EE | 2.727E-004 | 1 | 2.727E-004 | 94.64 | < 0.0001 | Significant |
|  | ζ | 0.90 | 1 | 0.90 | 1.42 | 0.2724 | Non-significant |
| A^2^ | PS | 2.741E-004 | 1 | 2.741E-004 | 108.65 | < 0.0001 | Significant |
|  | EE | 6.823E-006 | 1 | 6.823E-006 | 2.37 | 0.1677 | Non-significant |
|  | ζ | 97.97 | 1 | 97.97 | 154.06 | < 0.0001 | Significant |
| B^2^ | PS | 2.696E-007 | 1 | 2.696E-007 | 0.11 | 0.7533 | Non-significant |
|  | EE | 2.494E-005 | 1 | 2.494E-005 | 8.66 | 0.0216 | Significant |
|  | ζ | 11.62 | 1 | 11.62 | 18.27 | 0.0037 | Significant |
| C^2^ | PS | 1.761E-005 | 1 | 1.761E-005 | 6.98 | 0.0333 | Significant |
|  | EE | 1.094E-005 | 1 | 1.094E-005 | 3.80 | 0.0923 | Non-significant |
|  | ζ | 21.58 | 1 | 21.58 | 33.93 | 0.0006 | Significant |
| Residual | PS | 1.766E-005 | 7 | 2.523E-006 |  |  |  |
|  | EE | 2.017E-005 | 7 | 2.881E-006 |  |  |  |
|  | ζ | 4.45 | 7 | 0.64 |  |  |  |
| Lack of fit | PS | 1.155E-005 | 3 | 3.851E-006 | 2.52 | 0.1964 | Non-significant |
|  | EE | 1.233E-005 | 3 | 4.111E-006 | 2.10 | 0.2433 | Non-significant |
|  | ζ | 3.03 | 3 | 1.01 | 2.85 | 0.1691 | Non-significant |
| Pure Error | PS | 6.108E-006 | 4 | 1.527E-006 |  |  |  |
|  | EE | 7.838E-006 | 4 | 1.959E-006 |  |  |  |
|  | ζ | 1.42 | 4 | 0.35 |  |  |  |
| Cor Total | PS | 6.299E-003 | 16 |  |  |  |  |
|  | EE | 2.088E-003 | 16 |  |  |  |  |
|  | ζ | 421.79 | 16 |  |  |  |  |

**Table (S2): Model summary statistics for PS, EE and ζ.**

| Term | Description/value in PS | Description/value in EE | Description/value in ζ |
| --- | --- | --- | --- |
| Model order | Quadratic | Quadratic | Quadratic |
| Lambda (λ) | -0.5 | -0.5 | 1 |
| Transformation | Inverse square root | Inverse square root | None |
| R^2^ | 0.9960 | 0.9822 | 0.9871 |
| Adjusted R^2^ | 0.9936 | 0.9716 | 0.9770 |
| Predicted R^2^ | 0.9828 | 0.9280 | 0.9139 |
| Adequate precision | 62.01 | 32.841 | 32.349 |
| Equation | $\left( \mathrm{PS} \right)^{-0.5}=+0.12679+\left( 0.000728983A \right)-\left( 0.00101556B \right)+\left( 0.00110286C \right)+\left( 0.00033207AB \right)-\left( 0.00805531A^{2} \right)-\left( 0.0008129{08C}^{2} \right)$  Eq (7) | $\mathrm{EE}^{-0.5}= + 0.096834+\left( 0.021735A \right)-\left( 0.00149111B \right)+\left( 0.00232727C \right)-\left( 0.00161756AC \right)+\left( 0.000248314BC \right)-\left( 0.000554648B^{2} \right)$  Eq (8) | $\zeta=+25.68982+\left( 11.07875A \right)-\left( 0.79831B \right)-\left( 2.77050C \right)+\left( 0.27800AC \right)-\left( 4.82375A^{2} \right)+\left( 0.03756B^{2} \right)+\left( 0.090550C^{2} \right)$  Eq (9) |

**Table (S3): Effect of storage on particle size & association efficiency of SD/TPP_3_:CS-LPH_o_.**

| Time (month) | VMD  D[4,3]^a^ ±s.d | Span ±s.d | AE^b^ ±s.d  (%) |
| --- | --- | --- | --- |
| 0 | 4.947±0.89 | 2.160±0.38 | 97.63±3.28 |
| 1 | 5.533±0.43^ns^ | 1.555±0.55 | 96.89±1.43 ^ns^ |
| 3 | 5.876±0.67^ns^ | 1.457±0.03 | 97.67±2.78 ^ns^ |
| 6 | 6.206±0.10^*^ | 1.652±0.64 | 96.93±2.89 ^ns^ |

All results are expressed as mean of 3 determinations ±s.d, 0 month: freshly prepared.

^a^ VMD D[4,3]: particle size of dry powder measured using Mastersizer. ^b^ AE: association efficiency.

SD-M/TPP_3_:CS_8_-LPH_o_: spray dried TPP cross-linked CS coated LPH containing 1%w/v PLGA concentration, 15.39%w/w L/P concentration and 5%w/w MIR concentration, 0.2% w/v CS, 0.077%w/v Mann & 0.2%w/v TPP. CS-LPH_o_:TPP volume ratio was 2.5:1.

Statistical analysis was carried out comparing each dispersed SDP to the fresh state using *ANOVA* followed by *Tukey’s* multiple comparison test. ^ns^: non-significant (*p*>0.05) and ^*^: *p*<0.05.

**
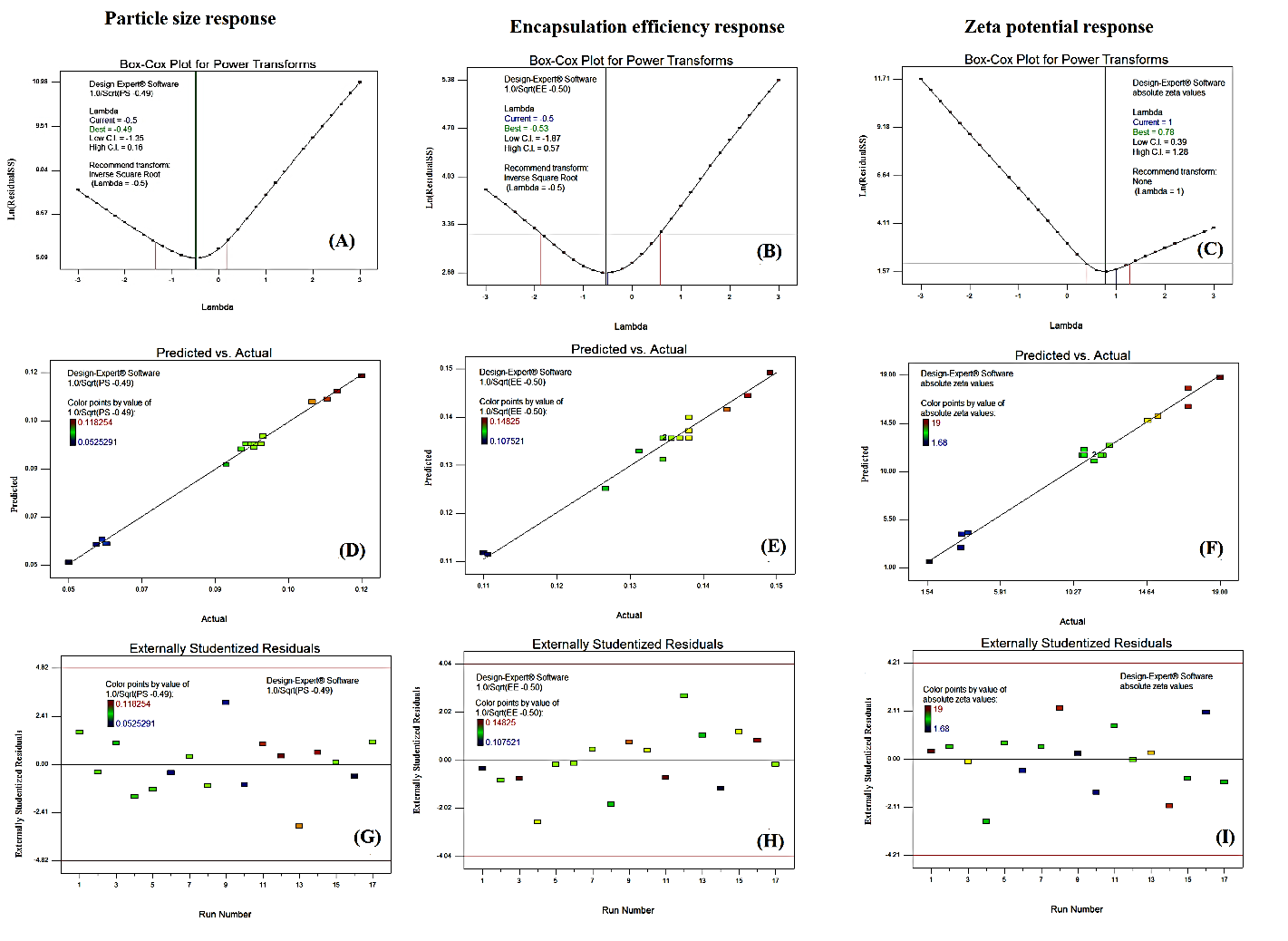
Fig. (S1) Plots of (A, B & C): “Box-Cox”, (D, E & F): “Predicted versus actual” and (G, H & I): “Externally Studentized Residuals” for PS, EE and ζ potential responses.**


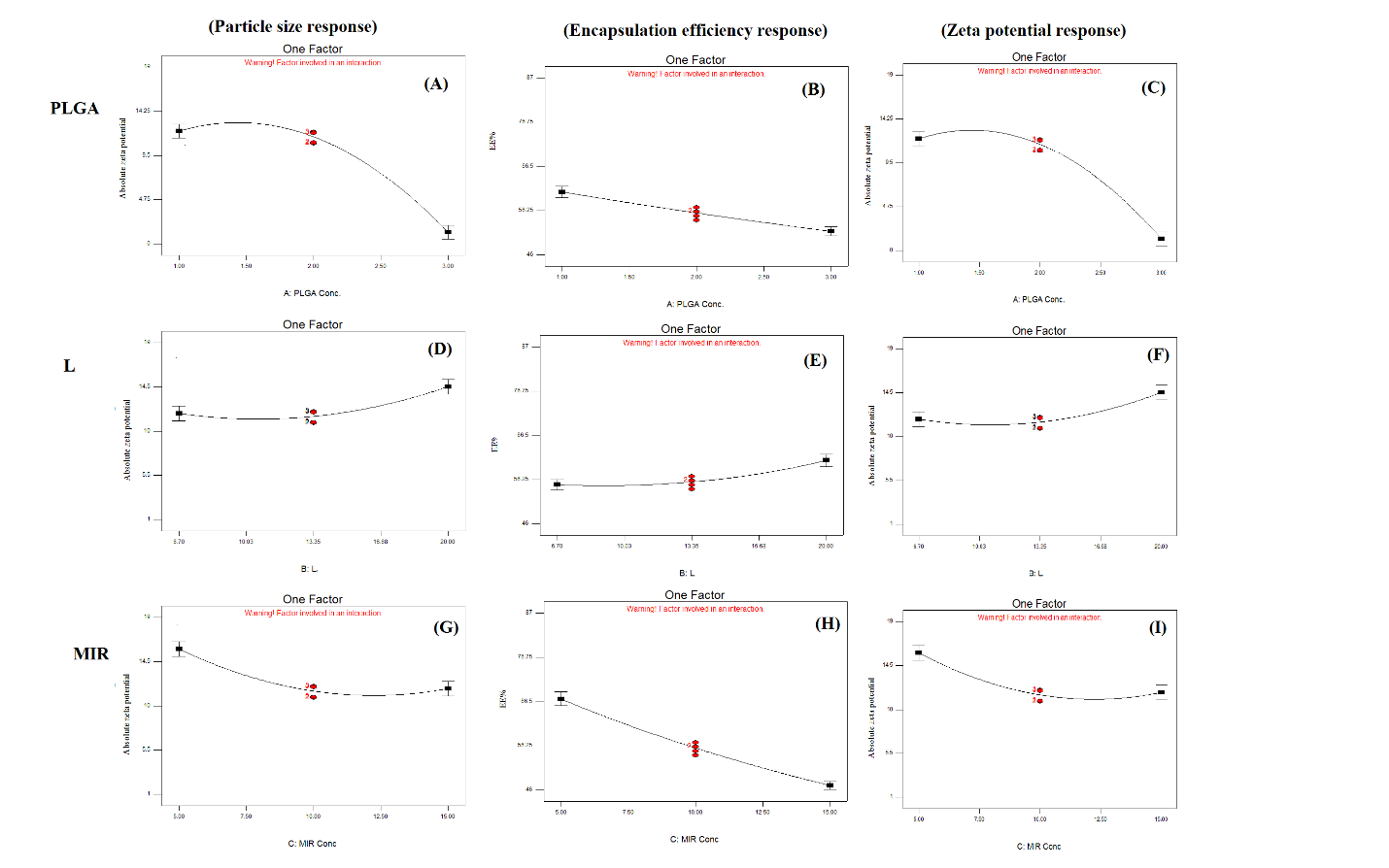
**Fig. (S2) Plot of PLGA, L, and MIR main effects on PS, EE% and ζ potential response.**
